# Investigation of end-stage kidney disease risk prediction in an ethnically diverse cohort of people with type 2 diabetes: use of kidney failure risk equation

**DOI:** 10.1136/bmjdrc-2024-004282

**Published:** 2024-09-13

**Authors:** Aicha Goubar, Anastasios Mangelis, Stephen Thomas, Nikolaos Fountoulakis, Julian Collins, Salma Ayis, Janaka Karalliedde

**Affiliations:** 1 Population Health Sciences, School of Life Course and Population Sciences, King's College London, London, UK; 2 King’s Health Partners and School of Cardiovascular Medicine & Sciences, King's College London, London, UK; 3 King's College Hospital NHS Foundation Trust, King's College London, London, UK; 4 King’s Health Partners and British Heart Foundation Centre of Excellence, School of Cardiovascular & Metabolic Medicine and Sciences, King's College London, London, UK

**Keywords:** Diabetes Mellitus, Type 2, Kidney Failure, Chronic, Risk Assessment

## Abstract

**ABSTRACT:**

**Introduction:**

The four variable kidney failure (KF) risk equation (KFRE) is recommended to estimate KF risk (ie, need for dialysis or kidney transplantation). Earlier referral to clinical kidney services for people with high-risk of kidney failure can ensure appropriate care, education and support are in place pre-emptively. There are limited data on investigating the performance of KFRE in estimating risk of end-stage kidney disease (ESKD) in people with type 2 diabetes mellitus (T2DM) and chronic kidney disease (CKD). The primary ESKD endpoint event was defined as estimated glomerular filtration rate (eGFR) <10 mL/min/1.73 m^2^ and secondary endpoint eGFR <15 mL/min/1.73 m^2^.

**Research design and methods:**

We studied 7296 people (30% women, 41% African-Caribbean, 45% Caucasian) with T2DM and CKD (eGFR median (range) 48 (15–59) mL/min/1.73 m^2^) were included at two hospitals in London (median follow-up 10.2 years). Time to ESKD event was the endpoint and Concordance index (C-index) was used to assess KFRE’s discrimination of those experiencing ESKD from those who did not. Mean (integrated calibration index (ICI)) and 90th percentile (E90) of the difference between observed and predicted risks were used as calibration metrics.

**Results:**

Of the cohort 746 (10.2%) reached ESKD primary event (135 (1.9%) and 339 (4.5%) over 2 and 5 years, respectively). Similarly, 1130 (15.5%) reached the secondary endpoint (270 (3.7%) and 547 (7.5%) over 2 and 5 years, respectively). The C-index for the primary endpoint was 0.842 (95% CI 0.836 to 0.848) and 0.816 (95% CI 0.812 to 0.820) for 2 and 5 years, respectively. KFRE ‘under-predicted’ ESKD risk overall and by ethnic group. Likewise, the C-index for secondary endpoint was 0.843 (0.839–0.847) and 0.801 (0.798–0.804) for 2 and 5 years, respectively. KFRE performance analysis performed more optimally with the primary endpoint with 50% enhancement of the calibration metrics than with the secondary endpoint. KFRE recalibration improved ICI by 50% and E90 by more than 78%.

**Conclusions:**

Although derived for predicting KF, KFRE also demonstrated good discrimination for ESKD outcome. Further studies are needed to identify variables/biomarkers that may improve KFRE’s performance/calibration and to aid the development of other predictive models to enable early identification of people at risk of advanced stages of CKD prior to onset of KF.

**WHAT IS ALREADY KNOWN ON THIS TOPIC**

- Kidney failure equation (KFRE) is the most well-validated tool recommended to predict the 2 and 5-year risk of progression to kidney failure (defined as need for dialysis or kidney transplantation) in patients with chronic kidney disease (CKD) stages 3A-5.

**WHAT THIS STUDY ADDS**

- We demonstrated that KFRE remains a useful tool to use for predicting progression to end-stage kidney disease defined by either estimated glomerular filtration rate <10 or <15 mL/min/1.73 m^2^ in people with type 2 diabetes.

**HOW THIS STUDY MIGHT AFFECT RESEARCH, PRACTICE OR POLICY**

- Further studies are needed to develop further predictive risk equations and/or identify variables/biomarkers that can improve the performance of KFRE in identifying those at highest risk of progression to advanced stages of CKD in type 2 diabetes.

## Introduction

 Chronic kidney disease (CKD) is a global health problem with increasing prevalence. CKD is associated with increased healthcare utilisation and costs as well as enhanced morbidity and mortality.

Most patients with CKD do not progress to end-stage kidney disease (ESKD), but it is vital to identify those at high risk of progression to ESKD to enable advanced clinical care planning and communicate with patient/family education and counsel them on the impact of ESKD. Indeed, the number of people reaching ESKD is increasing worldwide and will have major impact on global healthcare systems. It is, therefore, essential to have strategies to identify those at highest risk to enable prioritised care and access to specialist renal services. A multifactorial approach can slow progression of CKD and reduce related enhanced cardiovascular disease (CVD) morbidity and risk.

The four variable kidney failure risk equation (KFRE) is recommended to estimate the risk of kidney failure (KF) defined as need for dialysis or kidney transplantation and remains the most well-validated risk prediction tool, predicting the 2-year and 5-year risk of progression to KF in patients with CKD stages 3A-5.^1 2^ The four-variable KFRE incorporates age, sex, estimated glomerular filtration rate (eGFR) calculated by the Chronic Kidney Disease Epidemiology Collaboration formula (CKD-EPI)^3^ and urine albumin-creatinine ratio (ACR) to predict the need for kidney replacement and transplantation within 2-year and 5-year time frames. The four-variable KFRE has been demonstrated to be accurate for risk prediction for KF. Healthcare systems have utilised thresholds of risk such as a 2-year KF risk of ≥40% to guide access to advanced kidney care services and planning for renal replacement therapies.

There are, however, limited studies that have evaluated the KFRE in exploring whether the equation can be used to predict ESKD defined by eGFR <10 or <15 mL/min/1.73 m^2^ before the onset of KF in specific causes of CKD such as diabetes. Diabetes remains the leading cause of CKD and ESKD in many countries worldwide.^4^ Indeed, diabetes per se increases the progression of ESKD and is also associated with greater competing risk of all cause and CVD mortality compared with people without diabetes and equivalent degree of CKD.^5 6^ Ethnicity can also increase the progression of CKD towards ESKD and some ethnic groups appear to have a predisposition towards faster progression to KF as compared with others.^7 8^

Our primary aim is to evaluate whether we can prove the concept of evaluating the performance of KFRE in predicting the risk of ESKD considered as the endpoint defined primarily by eGFR <10 mL/min/1.73 m^2^. The secondary objective was to investigate the performance with eGFR <15 mL/min/1.73 m^2^ as a secondary endpoint. We investigated this in a multiethnic cohort of people with type-2 diabetes mellitus (T2DM) and CKD stage 3A or greater.

## Research design and methods

### Population cohort

Clinical and demographic data were collected from 19 648 people with a clinical diagnosis of T2DM, as recorded in their primary care and eye screening records, who attended surveillance diabetes eye screening in southeast London between 2004 and 2018. This excludes any pregnancy and/or any documented history of non-diabetic kidney disease on hospital records. The local general population of southeast London is ethnically diverse, with >30% of people being of African Caribbean heritage.^9^ Exclusion criteria for this study were CKD-EPI eGFRs <15 and eGFRs ≥60 mL/min/1.73 m^2^ at baseline. After applying this exclusion criteria, 11 652 individuals were excluded (422 and 11 230 with a baseline eGFR <15 and eGFR ≥60 mL/min/1.73 m^2^, respectively). About 7296 individuals with T2DM were eligible to be included in our study with at least two eGFR measurements, including the baseline eGFR between 15 and 60 mL/min/1.73 m^2^.

Anonymised diabetes-related clinical and biochemical data covering a time span between 2004 and 2018 were collected from electronic patient records at two large teaching hospitals in London.

The following variables were available: date of birth, date of death (if applicable), sex, self-reported ethnicity, date of diabetes diagnosis, anthropometrics (weight and height), systolic and diastolic blood pressure (BP) and laboratory measurements of serum creatinine, ACR, Hemoglobin A1c (HbA1c), low-density lipoprotein (LDL cholesterol), high-density lipoprotein (HDL cholesterol), total cholesterol and triglycerides. We also collected diabetes eye screening status and results of retinopathy and maculopathy (RM) grade assessments (monoscopic fundus photography of dilated pupils with a nonmydriatic digital camera) using national diabetes eye screening classifications.^10^

To derive the baseline variables (eGFR and ACR), which are required in the KFRE as well as other variables (ie, systolic and diastolic BP), we considered the date of the first serum creatinine measurement for each patient as the date of entry into the study and extracted all other baseline values within a 2-year span. When no measurement is found within that span, values were considered missing. The follow-up duration was defined as the time from baseline to last available creatinine measurement or date of death.

Serum creatinine was measured at two central laboratories run by the same laboratory services provider using an isotope dilution mass spectrometry-traceable modified enzymatic method on the Roche 702 platform (Roche, Basel, Switzerland). ACR measured in urine samples were taken from routine clinical care using immunoturbidimetry for albumin and enzymatic method for creatinine Roche 702 platform (Roche, Basel, Switzerland). Serum creatinine values that were measured during acute hospital admissions were excluded.

We applied the CKD-EPI eGFR formula using patients’ serum creatinine data to calculate eGFRs, which has recently been validated for use in the KFRE.^11^ Urinary ACR was captured from the routine clinical laboratory data.

### Outcome

We defined the primary ESKD event as reaching an eGFR <10 mL/min/1.73 m^2^ with at least one subsequent sustained eGFR <10 mL/min/1.73 m^2^. The second confirmatory eGFR <10 mL/min/1.73 m^2^ was the next available eGFR measurement. Any individual with only one eGFR below 10 was not considered as event and thus was censored. The primary outcome was defined as time from baseline to the primary ESKD event, last eGFR measurement (last follow-up) or to death whatever occurs first. Similarly, we considered a secondary endpoint as time from baseline to reaching eGFR <15 mL/min/1.73 m^2^.

### Statistical analyses

In this paper, we used the UK recalibrated four-variable KFRE to calculate the predicted 2-year and 5-year ESKD risk.^12^ ACR at baseline was available for 81% of individuals. We performed a Multiple Chained Equation imputation process to replace missing ACR data using the KFRE’s four variables in addition to auxiliary variables,^13^ including ethnicity, diastolic and systolic BP, HbA1c, triglycerides, RM, weight, BMI, HDL, LDL and total cholesterol to minimise bias and optimise power of the imputations. Forty distinct datasets were generated for efficient and stable estimates based on 40% overall missingness rate.^14^ Missing values were replaced iteratively with values using predictive mean matching approach.^15^

We evaluated the performance of the KFRE in predicting ESKD risk (using the primary and the secondary ESKD endpoint definitions, respectively) for 2-year and 5-year follow-up in terms of discrimination and calibration using relevant key performance measures of survival prediction models.^16^ KFRE discriminative ability of how it differentiates individuals who reached the event from those who did not was assessed by Harrell’s Concordance index (C-index)^17^ and Uno’s censoring adjusted C-index.^18^ To illustrate the discrimination’s strength, Kaplan-Meier curves were presented to describe ESKD-free probabilities over time within risk groups defined by the original proposed risk group definition^2^ (risk at 5-year prediction of ESKD <3%, 3% to <5%, 5% to <15%, 15% to <25%, 25% to <50% and ≥50%).

The calibration represents the agreement between the observed and predicted risks and was illustrated graphically within ten risk groups defined by tenth of predicted risks at 2 and 5 years, respectively. The calibrations for 2 and 5 years of follow-up were assessed using the complementary log–log transformed predicted risk included with a flexible functional form as the predictor within a secondary model^19^ of the outcome. A good calibration should be reflected by a perfect matching between the predicted and observed risks. We also used the distribution of the absolute difference (AD) between smoothed observed and predicted risks to summarise some calibration metrics. These metrics were the integrated calibration index (ICI: the average of ADs) and the E50 and E90 (the ADs median and 90th percentile, respectively).^19^ Bootstrap 95% CI of these calibration metrics were calculated using non-parametric bootstrap on 500 samples with replacement.

The risk predictions from the 40 imputed datasets were used to generate an average risk for every individual. We used these individual risk average to construct the groups defined earlier in the discrimination and calibration assessment.

Sensitivity analyses consisted of performing the investigation of KFRE using both the primary and secondary endpoints in the complete case analysis (CCA) dataset excluding individuals with missing ACR (19%). The data of the three other KFRE variables (age, eGFR and sex) were initially available for all individuals.

We also performed a recalibration of the original KFRE^2^ using the primary outcome’s survival function from the current cohort to adjust the baseline risk part in the KFRE (section 4 in online supplemental file).

All the above analyses were also carried out in Caucasian and African Caribbean groups respectively.

All data analyses were conducted in R V.4.2.0.^20^

## Results

Table 1 shows the baseline characteristics and clinical features of the 7296 people with T2DM and CKD and compares those who reached ESKD primary event to those who did not. Comparison of those who reached the secondary event with those who did not is also presented in table 1. Overall, the mean (SD) age was 65 (12) years old, 2955 (41%) were African-Caribbean, 3298 (45%) Caucasian and 1043 (14%) other ethnicities. Median (first quartile, third quartile) eGFR at baseline was 47.6 (38.2, 54.2) mL/min/1.73 m^2^ and ranges from 15 to 59 mL/min/1.73 m^2^.

**Table 1:** Baseline demographic and biochemical features of 7296 people with T2DM and CKD

	Overall (N=7296)	Reached eGFR<10 (N=746)	Did not reach eGFR≥10 (N=6550)	Reached eGFR<15 (N=1130)	Did not reach eGFR<15 (N=6166)
Age (years)					
Mean (SD)	64.9 (11.9)	63.3 (11.9)	65.1 (11.9)	65.1 (11.5)	64.9 (12.0)
Median (Q1, Q3)	66.0 (57.0, 73.0)	65.0 (56.0, 72.0)	66.0 (57.0, 74.0)	67.0 (57.2, 73.0)	66.0 (57.0, 73.0)
Ethnicity					
African Caribbean	2955 (40.5%)	289 (38.7%)	2666 (40.7%)	448 (39.6%)	2507 (40.7%)
Caucasian	398 (45.2%)	326 (43.7%)	2972 (45.4%)	508 (45.0%)	2790 (45.2%)
Asian	592 (8.1%)	69 (9.2%)	523 (8.0%)	107 (9.5%)	485 (7.9%)
Mixed	199 (2.7%)	30 (4.0%)	169 (2.6%)	30 (2.7%)	169 (2.7%)
Other	252 (3.5%)	32 (4.3%)	220 (3.4%)	37 (3.3%)	215 (3.5%)
Gender					
Female	2209 (30.3%)	238 (31.9%)	1971 (30.1%)	378 (33.5%)	1831 (29.7%)
Male	5087 (69.7%)	508 (68.1%)	4579 (69.9%)	752 (66.5%)	4335 (70.3%)
ACR (mg/mmol)					
Missing	1366	213	1153	297	1069
Mean (SD)	46.3 (45.7)	65.3 (61.6)	44.4 (43.4)	62.8 (58.5)	43.6 (42.7)
Median (Q1, Q3)	40.0 (18.7, 45.0)	41.0 (33.0, 60.7)	40.0 (17.0, 45.0)	41.0 (33.0, 63.6)	40.0 (16.4, 45.0)
ACR (mg/mmol)*					
Mean (SD)	46.8 (41.6)	62.9 (52.9)	44.9 (39.8)	60.8 (50.9)	44.2 (39.2)
Median (Q1, Q3)	41.0 (27.6, 47.0)	43.0 (36.0, 65.3)	41.0 (25.0, 46.0)	43.0 (36.0, 65.0)	41.0 (24.0, 46.0)
eGFR (mL/min/1.73 m^2^)					
Mean (SD)	45.2 (10.9)	36.6 (12.5)	46.2 (10.3)	38.1 (12.4)	46.5 (10.1)
Median (Q1, Q3)	47.6 (38.2, 54.2)	36.6 (26.1, 46.7)	48.4 (39.8, 54.5)	38.2 (27.9, 48.1)	48.7 (40.3, 54.6)
eGFR stage					
G3a	4226 (57.9%)	210 (28.2%)	4016 (61.3%)	369 (32.7%)	3857 (62.6%)
G3b	2203 (30.2%)	272 (36.5%)	1931 (29.5%)	408 (36.1%)	1795 (29.1%)
G4	867 (11.9%)	264 (35.4%)	603 (9.2%)	353 (31.2%)	514 (8.3%)

eGFR—using the Chronic Kidney Disease (CKD) Epidemiology Collaboration equation.

* For the 1366 (19%) individuals with missing ACR, data imputation was carried out to complete their data which was replaced by the average over 40 imputed datasets for every individual.

ACR, albumin-to-creatinine ratio; CVD, cardiovascular disease; eGFR, estimated glomerular filtration rate; ESKD, end-stage kidney disease; KFRE, kidney failure risk equation; Q1, first quartile defined by the 25th percentile; Q3, third quartile defined by the 75th percentile; T2DM, type 2 diabetes mellitus.

Of the cohort, 746 (10.2%) reached the primary endpoint with 135 (1.9%) over 2 years and 339 (4.6%) over 5 years. The median follow-up was 10.2 years. 1130 (15.5%) people reached the secondary endpoint with 270 (3.7%) over 2 years and 547 (7.5%) over 5 years. There were 674 (9%) individuals who had no confirmatory eGFR measurement <15 mL/min/1.73 m^2^ at baseline and were, therefore, censored. The baseline characteristics of our study cohort, of the UK cohort used in the UK-recalibrated KFRE^12^ and of the UK-cohorts^2^ used in the development of KFRE are presented in the additional file (online supplemental table S1). In the current study cohort, the proportion who reached ESKD primary endpoint is similar in the three ethnicity groups (9.8%, 9.9% and 12.6% in African-Caribbean, Caucasian and other ethnicities, respectively) as shown in the additional file (online supplemental table S2). Similarly, the proportion with secondary event in the ethnicity groups was similar to that in the whole cohort (online supplemental table S2).

### KFRE discrimination

**Figure 1:**
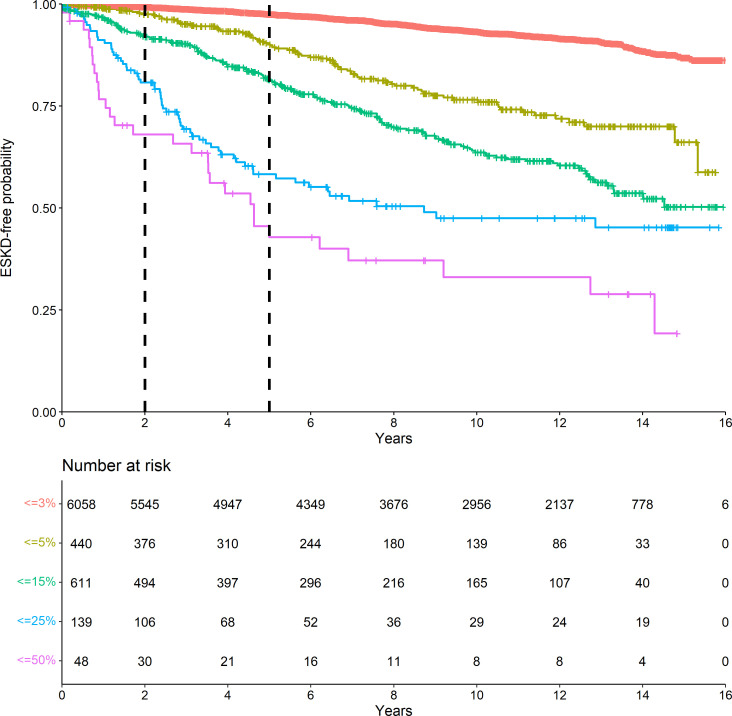
Kaplan-Meier curves of ESKD-free probabilities within original risk groups. Risk groups were defined as in the original KFRE development paper (5 years predicted risk<3%, 3% to<5%, 5% to<15%, 15% to<25%, 25% to<50% and ≥50%. ESKD, end-stage kidney disease; KFRE, kidney failure risk equation.

The discrimination ability of the KFRE in predicting ESKD risk for 2-year and 5-year follow-up was assessed with a C-index 0.843 (95% CI 0.839 to 0.847) for 2 years and 0.801 (95% CI 0.798 to 0.804) for 5 years) (table 2). By adjusting for censoring, the KFRE discrimination did not change, nor these findings change when we performed our investigation in African Caribbean and Caucasian separately (online supplemental table S3). Figure 1 does not suggest an adequate distinction between any two subsequent risk groups apart from the lowest risk group (<3%), which has the highest ESKD-free probability. Similar patterns were observed for both ethnic groups (online supplemental figure S2A).

**Table 2:** KFRE performance in the current study cohort including individuals with T2DM and CKD

	2 years			5 years		
	Discrimination		Calibration	Discrimination		Calibration
	Harrell’C-index	Uno’C-index	ICI, E50, E90 (95% CI)*	Harrell’C-index	Uno’C-index	ICI, E50, E90 (95% CI)*
Primary event defined as: EGFR<10 mL/min						
Full cohort†	0.842 (0.836, 0.848)	0.842 (0.836, 0.848)	0.014 (0.010, 0.017)	0.816 (0.812, 0.820)	0.816 (0.807, 0.816)	0.034 (0.029, 0.039)
			0.009 (0.007, 0.010)			0.023 (0.019, 0.028)
			0.030 (0.022, 0.038)			0.073 (0.058, 0.088)
Sensitivity analysis based on the CCA	0.854 (0.813, 0.895)	0.852 (0.810, 0.895)	0.011 (0.008, 0.015)	0.822 (0.793, 0.850)	0.816 (0.786, 0.846)	0.027 (0.022, 0.033)
			0.006 (0.005, 0.008)			0.017 (0.014, 0.021)
			0.026 (0.019, 0.034)			0.060 (0.045, 0.074)
Secondary event defined as: EGFR<15 mL/min						
Full cohort†	0.843 (0.839, 0.847)	0.842 (0.838, 0.846)	0.0338 (0.029, 0.039)	0.801 (0.798, 0.804)	0.793 (0.790, 0.800)	0.066 (0.059, 0.073)
			0.022 (0.018, 0.027)			0.049 (0.040, 0.057)
			0.073 (0.061, 0.084)			0.138 (0.120, 0.156)
Sensitivity analysis based on the CCA	0.844 (0.815, 0.874)	0.843 (0.813, 0.872)	0.031 (0.027, 0.035)	0.799 (0.776, 0.822)	0.790 (0.767, 0.814)	0.059 (0.051, 0.066)
			0.020 (0.018, 0.023)			0.042 (0.036, 0.048)
			0.069 (0.058, 0.080)			0.125 (0.109, 0.144)

E50, E90 the median and the 90th percentile of ADs.

ICI is the mean of absolute difference (AD) between observed and predicted risk.

* Non-parametric bootstrap 95% cCI of each of the ICI, E50 and E90 separately.

† Data imputation was carried out and results were summarised.

CCA, Complete Case Analysis dataset; CKD, chronic kidney disease; ICI, integrated calibration index; KFRE, kidney failure risk equation; T2DM, type 2 diabetes mellitus.

KFRE discrimination in our study within the CCA was comparable to that in the main analysis. Full findings of these analyses are in table 2, online supplemental table S4, online supplemental figures S5A,B and S7A,B.

Using the ESKD secondary event with threshold 15 mL/min, similar discrimination results were observed as with the primary definition in the whole cohort (table 2) as well as for both ethnic groups (online supplemental table S3, online supplemental figures S1 and S2B).

### KFRE calibration

**Figure 2:**
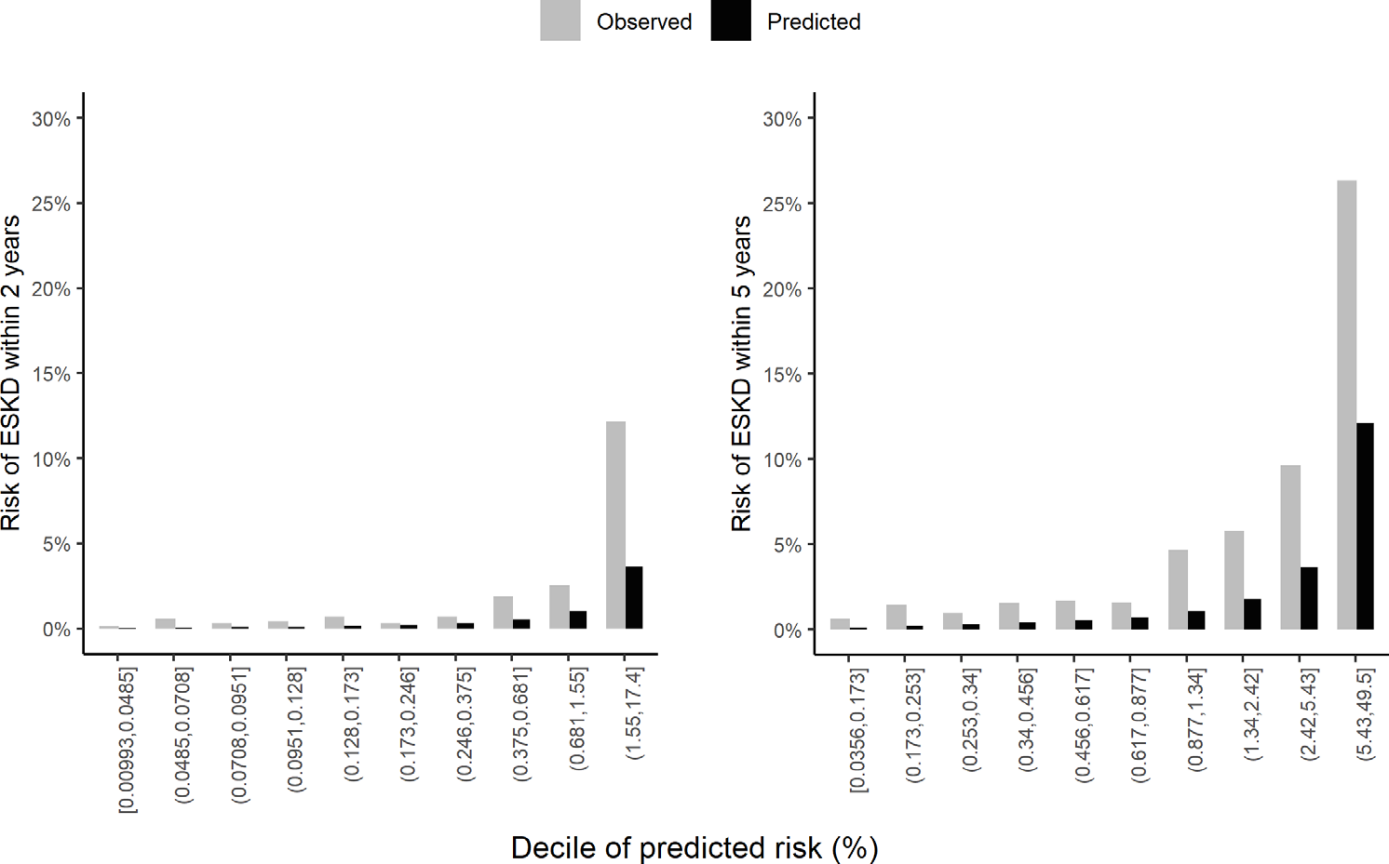
Observed and predicted ESKD risks in 10 groups. The 10 groups were defined by decile of predicted ESKD risks for 2-year and 5-year follow-up, respectively. ESKD, end-stage kidney disease.

The predicted risk of ESKD using the KFRE compared with the observed outcome is shown in figure 2 for the 2 and 5 years of follow-up. Figure 2 suggests that the KFRE is underpredicting risks overall as well as in both ethnic groups (online supplemental table S3, online supplemental figure S4A). Based on the three-calibration metrics (ICI, E50 and E90), risk underprediction for 5 years was generally higher than that for 2 years (table 2). These metrics are higher in Caucasian than in African Caribbean and particularly the E90 for 5 years (0.078, 95% CI 0.057 to 0.100 and 0.057 95% CI 0.036 to 0.078 for Caucasian and African Caribbean, respectively) (online supplemental table S3).

With the threshold 15 mL/min defining the study event, we also observed an underprediction of the 2-year and 5-year risks, but it was more significant compared with what was found with the primary event definition (table 2 and online supplemental figure S3). These observations were also noted in both ethnic groups (online supplemental table S3 & online supplemental figure S4B.

KFRE calibration in our study within the CCA was comparable to that in the main analysis for both the primary and the secondary endpoints (table 2 and online supplemental table S4, online supplemental figures S6A,B and S8A,B.

### KFRE recalibration

The baseline survival decreased by more than 2% compared with that for the original KFRE (from 0.9878 to 0.9654 for 2-year risk prediction and from 0.9570 to 0.8998 for 5-year risk across the imputation datasets) (online supplemental table S5). Kaplan-Meier curves of the ESKD-free probabilities showed a better discrimination between the five risk groups overall and in each ethnic group (online supplemental figures S9 and S11). Recalibration of the original KFRE performed far better than the KFRE (UK-recalibrated) with more than 50% improvement in ICI, more than 38% in E50 and more than 80% in E90 (online supplemental table S6 and online supplemental figure S10). A slight underprediction was found in the 10th highest risk groups for 2-year model while this was slightly reversed for the 5-year model. Similar trends were found in African Caribbean group (online supplemental table S6 and online supplemental figure S12).

## Discussion

In a multiethnic urban cohort of people with T2DM and CKD (eGFR between 15 and 60 mL/min/1.73 m^2^), we observed that KFRE adequately predicted CKD progression to ESKD/KF defined primarily by reaching an eGFR <10 mL/min/1.73 m^2^ or secondarily by eGFR <15 mL/min/1.73 m^2^. In our investigational study using a panel of statistical methods, the KFRE discrimination was consistent with previous works on ESKD risk prediction. We noted that KFRE ‘under-predicted’ the risk of ESKD in all the risk groups we defined by decile of 2-year and 5-year predicted risks suggesting a consistent pattern. This ‘underprediction’ still exists and is poorer when considering ESKD event as a sustained eGFR <15 mL/min/1.73 m^2^.

Our observations suggest that although an adequate KFRE discrimination was obtained, greater calibration would be required for its application to predict ESKD whether defined as a sustained eGFR <10 or as a sustained eGFR <15 mL/min/1.73 m^2^. Our findings also suggest that recalibration using our cohort data improved the calibration performance of the original KFRE. All results extend previous observations and confirm that in ethnically diverse cohort of people with T2DM and CKD, the KFRE remains a useful tool to predict progression to KF.

‘Underprediction’ of ESKD by KFRE in our study maybe because for the effect of death as a competing risk. The KFRE was designed without incorporating death as a competing risk and this may have an impact although one would assume that this would lead to an overestimation of risk rather than our findings of underestimation. Another possible explanation for this maybe because we utilised ESKD as primary endpoint rather than KF. Another potential explanation of the underprediction of ESKD risk in our results may be due to the fact that the median eGFR in our cohort is much higher than those reported in recent external validation studies for KFRE where eGFR<15 mL/min/1.73 m^2^ and eGFR <20 mL/min/1.73 m^2^ were studied and concordantly rates of ESKD were higher (30%–64% reached ESKD for 2 and 5 years).^21 22^ A further explanation could be that we defined ESKD primarily as eGFR<10 mL/min/1.73 m^2^ and secondarily as eGFR<15 mL/min/1.73 m^2^ (at least two consecutive values, respectively), which may over represent ESKD risk compared with using initiation of kidney replacement therapy as a definition of ESKD. A sustained eGFR <10 or 15 mL/min/1.73 m^2^ as defining ESKD is well established and widely used and is a sign of a kidney getting close to failure or already failed. Although the analysis using endpoint of a sustained eGFR <10 mL/min/1.73 m^2^ led to a less significant ‘underprediction’, this is likely explained by the fact that this is closer to KF due to most people commencing kidney replacement therapy at this level of kidney function. The primary purpose of the KFRE is to identify and prioritise those at highest risk of progression to KF. In our opinion, investigating the criteria of a sustained eGFR <10 and <15 mL/min/1.73 m^2^ as outcomes in the KFRE is pragmatic and clinically relevant and was an attempt to prove the concept whether performance is similar as with the original KF outcome. This is based on the clinical rationale that earlier identification of people with high risk of ESKD being essential and more tools are needed to help avoid people presenting late with advanced CKD.

A recent large study^11^ of 59 cohorts has addressed some of the questions our study raised. The study aimed to validate the KFRE original equation ^2^ using the CKD-EPI 2021 creatinine equation for estimating GFR,^23^ as opposed to the CKD-EPI 2009 formula ^3^ used in the initial KFRE and found that CKD-EPI 2021 did not improve KFRE prediction performance, nor does additional variables and/or accounting for the competing risk of death. Additionally, the study highlighted heterogeneity in prediction performance across cohorts, identifying eight that substantially overpredicted 2-year risk and six that underpredicted it out of 58 cohorts. The 5-year risk on the other hand was overpredicted by three of 20 cohorts, but no under prediction was noted. The study has also shown substantial heterogeneity across subgroups. For example, underprediction of the 2-year risk was found in five out of eight cohorts in people with higher levels of eGFR (45–59 mL/min/1.73 m^2^), and over-prediction among those age 65+, in four cohorts out of eight. These two examples were among other 10 subgroups where large deviation in calibration was noted.

Our study has several strengths. First, it included a large sample size, along with a high event rate, from a heterogeneous ethnically diverse urban population with T2DM and CKD with baseline eGFR <60 mL/min/1.73 m^2^. Baseline eGFR was higher than many studies to date and this is clinically relevant as KFRE utility is to identify those at high risk of progression and to refer people at high risk early to dedicated renal services. Our data were collected in a real-world setting as part of routine clinical care and at two large university hospitals where standardised laboratory methodology was used.

We acknowledge several limitations. Comparison of KFRE predictive performance was done only in people with T2DM and CKD with at least one eGFR between 15 and 60 mL/min/1.73 m^2^ at baseline and we used ESKD rather than KF as primary endpoint. The majority (86%) of the study cohort had a second eGFR <60 mL/min/1.73 m^2^ within 2 years of their baseline and we knowledge the limitation due to including some individuals without a second confirmatory eGFR <60 mL/min/1.73 m^2^ in our analyses (14%). However, the baseline characteristics in this specific cohort (N=6249) are similar to that in the whole study cohort (the proportion with primary and secondary event was slightly higher (11.4% and 17.1%, respectively) (online supplemental table S7). In addition, post hoc analyses in this specific cohort showed that the KFRE performance are comparable to that in the whole study cohort (online supplemental table S8). We also knowledge the limitation due to using the endpoints as reaching eGFR <10 and 15 mL/min/1.73 m^2^, respectively, rather than KF per se. However, the aim of this study was to assess the utility of using KFRE to detect people with ESKD defined as eGFR <10 and <15 mL/min/1.73 m^2^. In addition, it has been recently shown that the accuracy of reporting KF might not be guaranteed in either primary or secondary care data due to issues in coding and therefore failure in identifying prevalent patients with KF or erroneously identification remains problematic.^24^ Therefore, our choice was to try assessing the utility of KFRE to identify people before KF needing treatment (which tends to occur around 8–9 mL/min in UK).

We cannot exclude the potential impact of immortal time bias as our study design was a retrospective observational cohort where we included people with a first eGFR measurement between 15 and 60 mL/min and who were attending routine clinical care at two large university hospitals in an ethnically diverse urban environment. We used this eGFR inclusion criteria as this was also utilised for the KFRE validation in previous studies. We also acknowledge that urine ACR missing data were imputed for 19% of people. We, however, performed further analyses excluding those with missing data (ie, CCA dataset) and observed results consistent with our main analyses.

Aetiology of CKD was defined based on the assessment by clinical records. Diabetic kidney disease was not proven by biopsy, and we cannot definitively conclude that the KFRE has comparable accuracy across the different kidney disease aetiologies. The clinical data of people attending two large hospitals in urban multiethnicity will limit the generalisability of the results to other clinical settings.

There are several important roles of the KFRE from identifying and referring people at high risk to specialised kidney clinics to communicating risk of ESKD and planning for renal replacement therapy. Objective equations such as KFRE are more accurate than subjective assessments of risk with data demonstrating KFRE is better than the predicted estimates from clinicians who tend to overestimate risk.

Prompt referral of those at highest need and risk is essential in resource scarce systems and using KFRE to triage has been proposed in recent guidelines. A KFRE risk cut-off of ≥10% at 2 years or >5% at 5 years has been proposed to identify and select patients into advanced care kidney clinics.^25^ Whether such approaches translate to better clinical outcomes remain unclear. However, clinical benefits of such preemptive/prompt review, education and assessment for high-risk individuals at risk of ESKD are likely.

In conclusion, in this study, with large ethnically diverse cohort of people with T2DM and CKD with eGFR <60 mL/min/1.73 m^2^, our investigation showed that the discrimination of KFRE between people experiencing ESKD from those who did not was good at 2 and 5 years, respectively. Further studies are needed to develop other predictive risk equations ^11^ and/or to identify variables/biomarkers that can improve the calibration and performance of KFRE and to development of other predictive models to enable early identification of people at the highest risk of progression to advanced stages of CKD prior to onset of KF.

## Supplementary material

10.1136/bmjdrc-2024-004282 online supplemental file 1

## Data Availability

No data are available.
