# Supplementary material for: Investigation of end-stage kidney disease risk prediction in an ethnically diverse cohort of people with type 2 diabetes: use of kidney failure risk equation
Source: BMJ Open Diabetes Res Care. 2024 Sep 13;12(4):e004282. doi: 10.1136/bmjdrc-2024-004282 (PMC11404155; doi:10.1136/bmjdrc-2024-004282)
Supplement: online supplemental file 1 [file bmjdrc-12-4-s001.docx]

**Additional Materials**

**Investigation of end stage kidney disease risk prediction in an ethnically diverse cohort of people with type 2 diabetes: Use of kidney failure risk equation.**

**Contents**

[**1. The study cohort baseline characteristics compared with development UK-cohorts** 4](#_Toc173423556)

[Table S1: Main baseline characteristics, follow-up, and outcomes of our study cohort compared to the previous UK-cohorts used in the KFRE development and in external validation. 4](#_Toc173423557)

[**2. Additional results from the primary analysis** 5](#_Toc173423558)

[Table S2: Baseline demographics and biochemical features of 7,296 people with T2DM and CKD by ethnicity group 5](#_Toc173423559)

[Table S3: KFRE performance in African-Caribbean and Caucasian with T2DM and CKD. 7](#_Toc173423560)

[Figure S1: Kaplan Meier curves of ESKD-free probabilities by risk group in the study cohort when the study event is defined by a sustained eGFR <15ml/min. Risk groups defined as in the original KFRE development paper (predicted risk <3%, 3% to <5%, 5% to <15%, 15% to <25%, 25% to <50% and >=50%. 9](#_Toc173423561)

[Figure S2: Kaplan Meier curves of ESKD-free probabilities by risk group in African Caribbean and Caucasian. Risk groups defined as in the original KFRE development paper (predicted risk <3%, 3% to <5%, 5% to <15%, 15% to <25%, 25% to <50% and >=50%. A: predictions based on the primary event defined by sustained eGFR<10ml/min. B: predictions based on secondary event defined by sustained eGFR<15ml/min 10](#_Toc173423562)

[Figure S3: Observed and predicted ESKD risks for 2 and 5 years in the full study cohort with secondary event defined as a sustained eGFR<15ml/min. Risks calculated in groups defined by decile of predicted risks. 11](#_Toc173423563)

[Figure S4: Observed and predicted ESKD risks for 2 and 5 years in African Caribbean and Caucasian. ESKD risks calculated in groups defined by decile of predicted risks. (A) with primary event defined as sustained eGFR<10 ml/min. (B) secondary event defined as sustained eGFR<15 ml/min. 12](#_Toc173423564)

[**3. Sensitivity analyses in the complete case analysis (CCA) dataset. Results from the primary and secondary analyses** 13](#_Toc173423565)

[Table S4: KFRE performance in African-Caribbean and Caucasian with T2DM and CKD. 13](#_Toc173423566)

[Figure S5: Kaplan Meier curves of ESKD-free probabilities by risk groups in the complete case analysis dataset. Risk groups defined as in the original KFRE development paper (predicted risk <3%, 3% to <5%, 5% to <15%, 15% to <25%, 25% to <50% and >=50%. A: predictions based on the primary event defined by sustained eGFR<10ml/min. B: predictions based on secondary event defined by sustained eGFR<15ml/min 15](#_Toc173423567)

[Figure S6: Observed and predicted ESKD risks for 2 and 5 years in the complete case analysis dataset. ESKD risks calculated in groups defined by decile of predicted risks. (A) with primary event defined as sustained eGFR<10 ml/min. (B) secondary event defined as sustained eGFR<15 ml/min 16](#_Toc173423568)

[Figure S7: Kaplan Meier curves of ESKD-free probabilities by risk groups in each ethnicity group in the complete case analysis dataset. Risk groups defined as in the original KFRE development paper (predicted risk <3%, 3% to <5%, 5% to <15%, 15% to <25%, 25% to <50% and >=50%. A: predictions based on the primary event defined by sustained eGFR<10ml/min. B: predictions based on secondary event defined by sustained eGFR<15ml/min 17](#_Toc173423569)

[Figure S8: Observed and predicted ESKD risks for 2 and 5 years in each ethnicity group in the complete case analysis dataset. ESKD risks calculated in groups defined by decile of predicted risks. (A) with primary event defined as sustained eGFR<10 ml/min. (B) secondary event defined as sustained eGFR<15 ml/min. 18](#_Toc173423570)

[**4. Recalibration of the original KFRE** 19](#_Toc173423571)

[Table S5: Baseline survival in the original KFRE, the UK recalibrated KFRE and in the current cohort of people with T2DM and CKD 19](#_Toc173423572)

[Table S6: Calibration metrics with recalibrated KFRE in the full cohort using the primary ESKD event. 20](#_Toc173423573)

[Figure S9: Kaplan Meier curves of ESKD-free probabilities by risk groups based on the recalibration of the original KFRE using the primary event. 21](#_Toc173423574)

[Figure S10: Observed and predicted primary ESKD risks for 2 and 5 years based on the recalibration of the original KFRE. ESKD risks calculated in groups defined by decile of predicted risks. 22](#_Toc173423575)

[Figure S11: Kaplan Meier curves of ESKD-free probabilities by risk groups in each ethnicity group based on the recalibration of the original KFRE using the primary event. 23](#_Toc173423576)

[Figure S12: Observed and predicted ESKD risks for 2 and 5 years in each ethnicity group based on the recalibration of the original KFRE using the primary event. ESKD risks calculated in groups defined by decile of predicted risks. 24](#_Toc173423577)

[**5. post-hoc analysis in the subgroup of individuals with an eGFR measurements<60ml/min within 2 years from the baseline** 25](#_Toc173423578)

[Table S7: Baseline demographic and biochemical features of 7,296 people with T2DM and CKD and in the subgroup with a confirmatory eGFR<60ml/min within two years from the baseline. 25](#_Toc173423579)

[Table S8: KFRE performance in the subgroup with an eGFR<60 within 2 years from the baseline. 26](#_Toc173423580)

[**References** 27](#_Toc173423581)

## **1. The study cohort baseline characteristics compared with development UK-cohorts**

Table S1: Main baseline characteristics, follow-up, and outcomes of our study cohort compared to the previous UK-cohorts used in the KFRE development and in external validation.

|  | **Current T2DM CKD cohort** | **UK-based cohort in PLOS Med study ^1^** | **UK-KFRE cohorts± ^2^** |
| --- | --- | --- | --- |
| **Baseline characteristics**† | 7 296 | 35 539 | 1 315¶ |
| Dates of baseline data | 2004-2018 | 2004–2016 | 1996–1998 and 2003‡ |
| Female | 2 209 (30.3%) | 20 436 (57.5%) | 757 (45.5%) |
| Ethnicity |  |  |  |
| African Caribbean | 2 955 (40.5%) | — | — |
| Caucasian | 3 298 (45.2%) | — | — |
| Other | 1 043 (14.3%) | — | — |
| Mean age, years | 64.9 (SD 11.9) | 75.9 (SD 10.6) | 68.2 |
| CKD-EPI eGFR, ml/min/1.73 m2 |  |  |  |
| Mean | 45.2 (SD 10.9) | 48.2 (SD 9.8) | 28.2 |
| Median | 47.6 (IQR 38.2 to 54.2) | 51 (IQR 43 to 56) | — |
| ACR, mg/mmol |  |  |  |
| Mean | 46.8 (SD 41.6) | 11.8 (SD 40.9) | — |
| Median | 41.0 (IQR 27.6 to 47.0) | 3.2 (IQR 1.2 to 8.0) | — |
| Participants with albuminuria§ | 6 961 (95.4%) | 17 546 (49.4%) | 960 (69.1%) |
| Cardiovascular disease | — | 11 376 (32.0%) | — |
| Heart failure | — | 3 191 (9.0%) | — |
| Hypertension | — | 24 833 (69.9%) | 988 (71.1%) |
| Diabetes | 7 296 (100%) | 11 193 (31.5%) | 670 (48.2%) |
| **Follow-up and outcomes** |  |  |  |
| Mean follow-up, years | 8.4 (SD 4.6) | 4.8 (SD 2.5) | — |
| Median follow-up, years | 8.9 (IQR 4.5 to 12.6) | 4.7 (IQR 2.8 to 6.6) | — |
| Mean time to ESRD, years | 5.6 (SD 4.0) | 3.5 (SD 2.3) | — |
| Median time to ESRD, years | 5.2 (IQR 2.1 to 8.5) | 3.3 (IQR 1.7 to 5.0) | — |
| ESRD events within 2 years* | 135 | 176 | — |
| ESRD events within 5 years* | 339 | 429 | 312 |
| ESRD rate, per 1,000 person-years | 4.3 (95% CI 4.0 to 4.7) | 3.4 (95% CI 3.1 to 3.6) | — |
| Death rate, per 1,000 person-years | — | 55.9 (95% CI 54.8 to 57.0) | 55.9 |

†Data are n (percent) unless otherwise indicated. ‡Dates refer to CRIB and GLOMMS-1, respectively.

**±**UK-based cohorts CRIB and GLOMMS-1 ^1^.

¶Overall, 308 of the 382 individuals in CRIB were used in the development of the 4-variable KFRE.

§Defined in KFRE development cohort as ACR ≥ 30 mg/g (≥3.39 mg/mmol).

*For our cohort figures correspond to the primary event defined as reaching eGFR<10ml/min/1.73m^2^

ACR, albumin-to-creatinine ratio; CKD, chronic kidney disease; CKD-EPI, Chronic Kidney Disease Epidemiology

Collaboration; eGFR, estimated glomerular filtration rate; ESRD, end stage renal disease as used KFRE development (CRIB and GLOMMS-1) ^1^ and in the external validation of KFRE ^2^. In the current study cohort ESKD was used instead as the event of interest.

## **2. Additional results from the primary analysis**

Table S2: Baseline demographics and biochemical features of 7,296 people with T2DM and CKD by ethnicity group

|  | **Total**  **(N=7,296)** | **African-Caribbean (N=2,955)** | **Caucasian (N=3,298)** | **Other**  **(N=1,043)** |
| --- | --- | --- | --- | --- |
| **ESKD primary event** | 746 (10.2%) | 289 (9.8%) | 326 (9.9%) | 131 (12.6%) |
| **ESKD Secondary event** | 1130 (15.5%) | 448 (15.2%) | 508 (15.4%) | 174 (16.7%) |
| **Age** |  |  |  |  |
| Mean (SD) | 64.9 (11.9) | 62.0 (12.4) | 67.9 (11.0) | 63.7 (11.0) |
| Median (Q1, Q3) | 66.0 (57.0, 73.0) | 64.0 (52.0, 71.0) | 69.0 (61.0, 76.0) | 65.0 (57.0, 71.0) |
| **Ethnicity** |  |  |  |  |
| African Caribbean | 2,955 (40.5%) | 2,955 (100%) |  |  |
| Caucasian | 3,298 (45.2%) |  | 3,298 (100%) |  |
| Asian | 592 (8.1%) |  |  | 592 (56.8%) |
| Mixed | 199 (2.7%) |  |  | 199 (19.1%) |
| Other | 252 (3.5%) |  |  | 252 (24.2%) |
| **Gender** |  |  |  |  |
| Female | 2,209 (30.3%) | 998 (33.8%) | 952 (28.9%) | 259 (24.8%) |
| Male | 5,087 (69.7%) | 1,957 (66.2%) | 2,346 (71.1%) | 784 (75.2%) |
| **Creatinine** |  |  |  |  |
| Mean (SD) | 117.4 (33.0) | 118.5 (32.3) | 116.7 (33.4) | 116.3 (33.4) |
| Median (Q1, Q3) | 107.0 (96.0, 126.0) | 108.0 (98.5, 127.0) | 105.0 (95.0, 127.0) | 106.0 (96.0, 123.0) |
| **ACR (mg/mmol)** |  |  |  |  |
| N-Miss | 1,366 | 444 | 736 | 186 |
| Mean (SD) | 46.3 (45.7) | 45.6 (43.3) | 45.0 (45.9) | 52.3 (51.6) |
| Median (Q1, Q3) | 40.0 (18.7, 45.0) | 41.0 (22.0, 45.0) | 39.0 (15.7, 45.0) | 41.0 (23.0, 46.0) |
| **ACR (mg/mmol)**† |  |  |  |  |
| Mean (SD) | 46.8 (41.6) | 45.9 (40.2) | 45.4 (40.8) | 53.4 (47.2) |
| Median (Q1, Q3) | 41.0 (27.6, 47.0) | 41.0 (28.8, 46.0) | 40.6 (25.0, 46.9) | 43.0 (30.9, 55.8) |
| **eGFR (ml/min/1.73 m^2^)** |  |  |  |  |
| Mean (SD) | 45.2 (10.9) | 45.4 (10.6) | 44.8 (11.2) | 46.0 (10.7) |
| Median (Q1, Q3) | 47.6 (38.2, 54.2) | 47.7 (39.0, 54.0) | 47.2 (37.2, 54.2) | 48.5 (39.2, 54.5) |
| **GFR stage** |  |  |  |  |
| G3a | 4,226 (57.9%) | 1,736 (58.7%) | 1,862 (56.5%) | 628 (60.2%) |
| G3b | 2,203 (30.2%) | 898 (30.4%) | 998 (30.3%) | 307 (29.4%) |
| G4 | 867 (11.9%) | 321 (10.9%) | 438 (13.3%) | 108 (10.4%) |

SD: standard deviation, Q1: 1^st^ quartile defined by the 25^th^ percentile, Q3: 3^rd^ quartile defined by the 75^th^ percentile. ACR, albumin-to-creatinine ratio; eGFR, estimated glomerular filtration rate using the Chronic Kidney Disease (CKD) Epidemiology Collaboration equation; ESKD, end stage kidney disease.

†For the 1,366 (19%) individuals (444 (15%), 736 (22%) & 186 (18%) in African-Caribbean, Caucasian and other subgroup ethnicity) with missing ACR data imputation was carried out to complete their data which was replaced by the average over 40 imputed datasets for each individual.

Table S3: KFRE performance in African-Caribbean and Caucasian with T2DM and CKD.

|  | **2 years** | | | | **5 years** | | |
| --- | --- | --- | --- | --- | --- | --- | --- |
|  | **Discrimination** | | **Calibration** | **Discrimination** | | | **Calibration** |
|  | **Harrell’C-index** | **Uno’C-index** | **ICI, E50, E90**  **(95% CI) )^¶^** | **Harrell’C-index** | | **Uno’C-index** | **ICI, E50, E90**  **(95% CI)^¶^** |
| **Primary event defined as: EGFR<10 ml/min** |  |  |  |  | |  |  |
| **African-Caribbeans** | 0.852  (0.844, 0.860) | 0.843  (0.845, 0.861) | 0.013  (0.007, 0.018)  0.009  (0.005, 0.012)  0.028  (0.015, 0.041) | 0.832  (0.826, 0.838) | | 0.831  (0.825, 0.838) | 0.027  (0.020, 0.035)  0.021  (0.015, 0.027)  0.057  (0.036, 0.078) |
| **Caucasian** | 0.827  (0.817, 0.837) | 0.823  (0.814, 0.833) | 0.013  (0.008, 0.019)  0.008  (0.005, 0.011)  0.029  (0.016, 0. 042) | 0.800  (0.794, 0.807) | | 0.792  (0.785, 0.799) | 0.036  (0.028, 0.045)  0.024  (0.018, 0.030)  0.078  (0.057, 0.100) |
| **Secondary event defined as: EGFR<15 ml/min** |  |  |  |  | |  |  |
| **African-Caribbeans** | 0.840  (0.834, 0.846) | 0.839  (0.833, 0.846) | 0.031  (0.023, 0.039)  0.021  (0.016, 0.027)  0.065  (0.046, 0.084) | 0.806  (0.801, 0.812) | | 0.802  (0.800, 0.808) | 0.056  (0.046, 0.065)  0.044  (0.034, 0.053)  0.112  (0.085, 0.138) |
| **Caucasian** | 0.849  (0.843, 0.855) | 0.846  (0.841, 0.852) | 0.0371  (0.029, 0.046)  0.023  (0.017, 0.028)  0.084  (0.062, 0.105) | 0.803  (0.800, 0.808) | | 0.791  (0.786, 0.797) | 0.076  (0.064, 0.087)  0.051  (0.040, 0.062)  0.167  (0.137, 0.200) |

*Data imputation was carried out and results were summarised. ICI: The integrated calibration index, the mean of Absolute Difference (AD) between observed and predicted risk. E50, E90 the median and the 90th percentile of ADs. ^¶^ non-parametric bootstrap 95% confidence limits of each of the ICI, E50 and E90 separately.

Figure S1: Kaplan Meier curves of ESKD-free probabilities by risk group in the study cohort when the study event is defined by a sustained eGFR <15ml/min. Risk groups defined as in the original KFRE development paper (predicted risk <3%, 3% to <5%, 5% to <15%, 15% to <25%, 25% to <50% and >=50%.


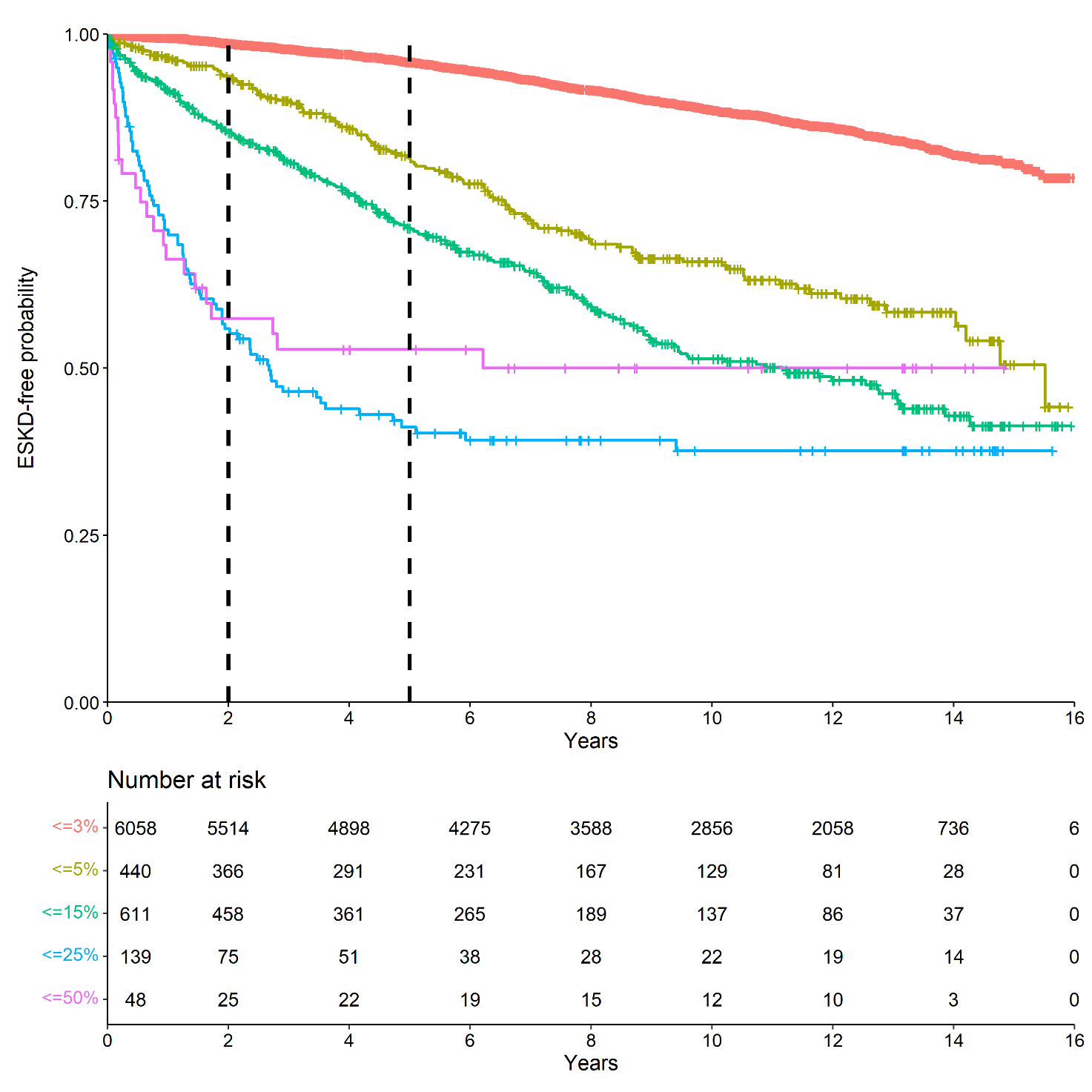


Figure S2: Kaplan Meier curves of ESKD-free probabilities by risk group in African Caribbean and Caucasian. Risk groups defined as in the original KFRE development paper (predicted risk <3%, 3% to <5%, 5% to <15%, 15% to <25%, 25% to <50% and >=50%. A: predictions based on the primary event defined by sustained eGFR<10ml/min. B: predictions based on secondary event defined by sustained eGFR<15ml/min


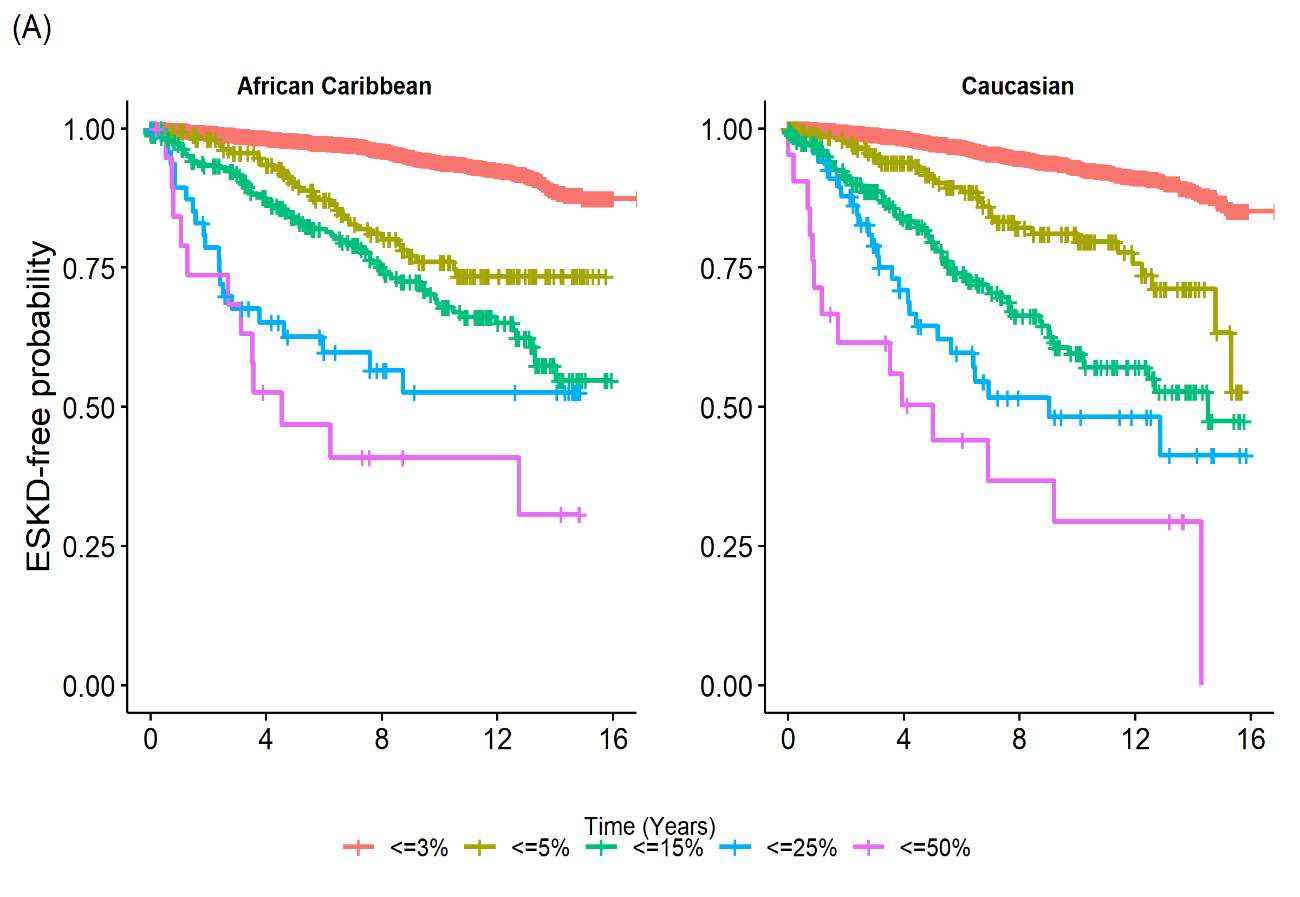


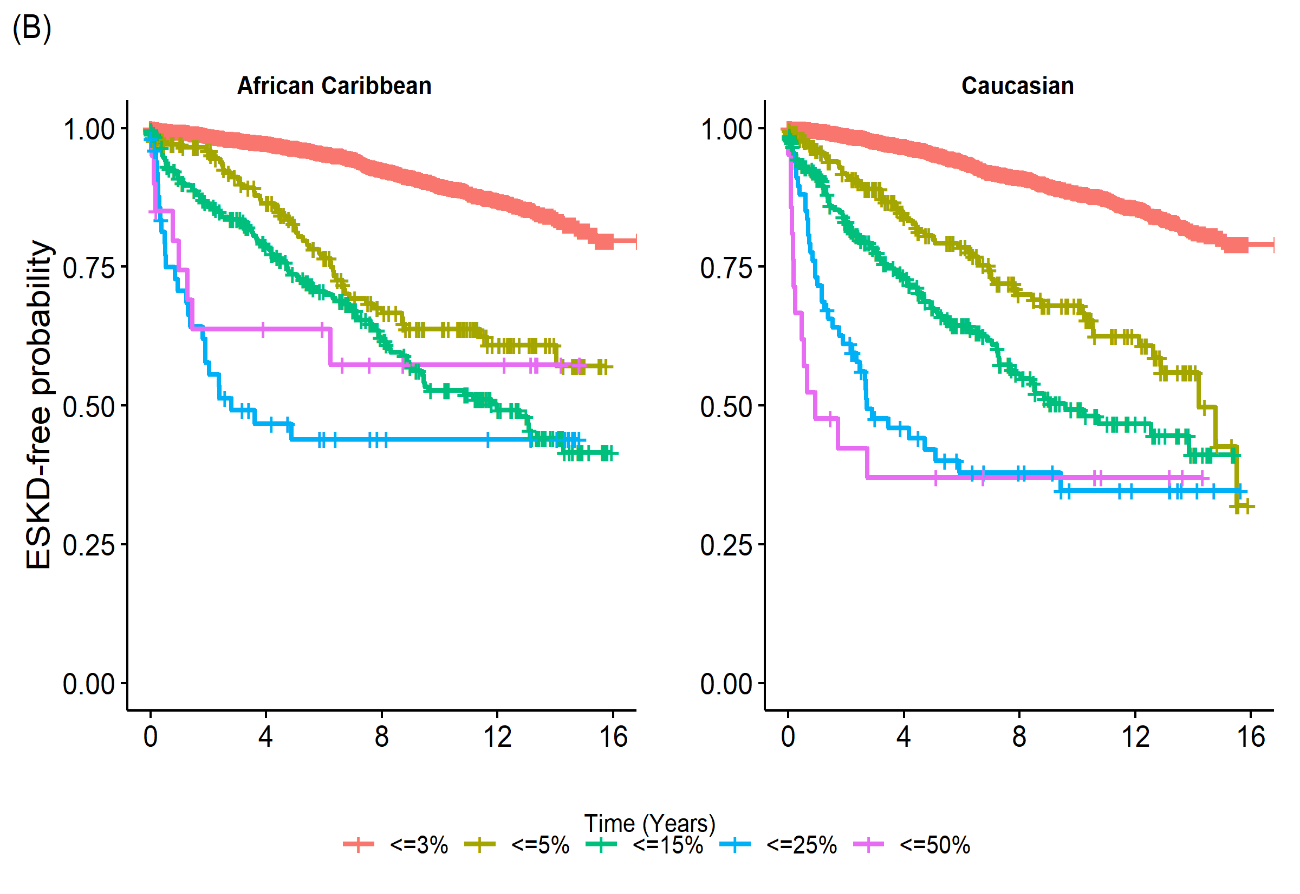


Figure S3: Observed and predicted ESKD risks for 2 and 5 years in the full study cohort with secondary event defined as a sustained eGFR<15ml/min. Risks calculated in groups defined by decile of predicted risks.


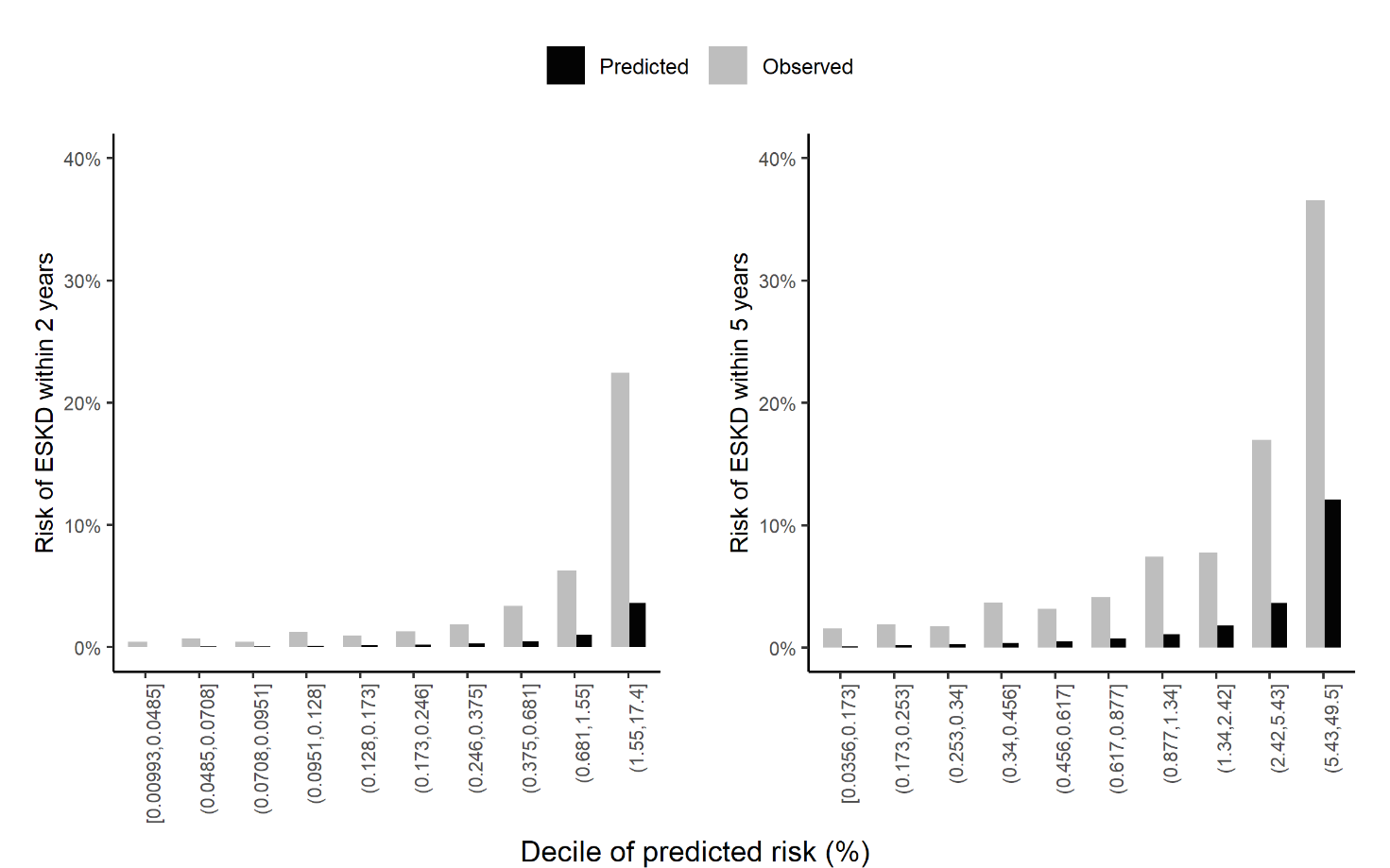


Figure S4: Observed and predicted ESKD risks for 2 and 5 years in African Caribbean and Caucasian. ESKD risks calculated in groups defined by decile of predicted risks. (A) with primary event defined as sustained eGFR<10 ml/min. (B) secondary event defined as sustained eGFR<15 ml/min.


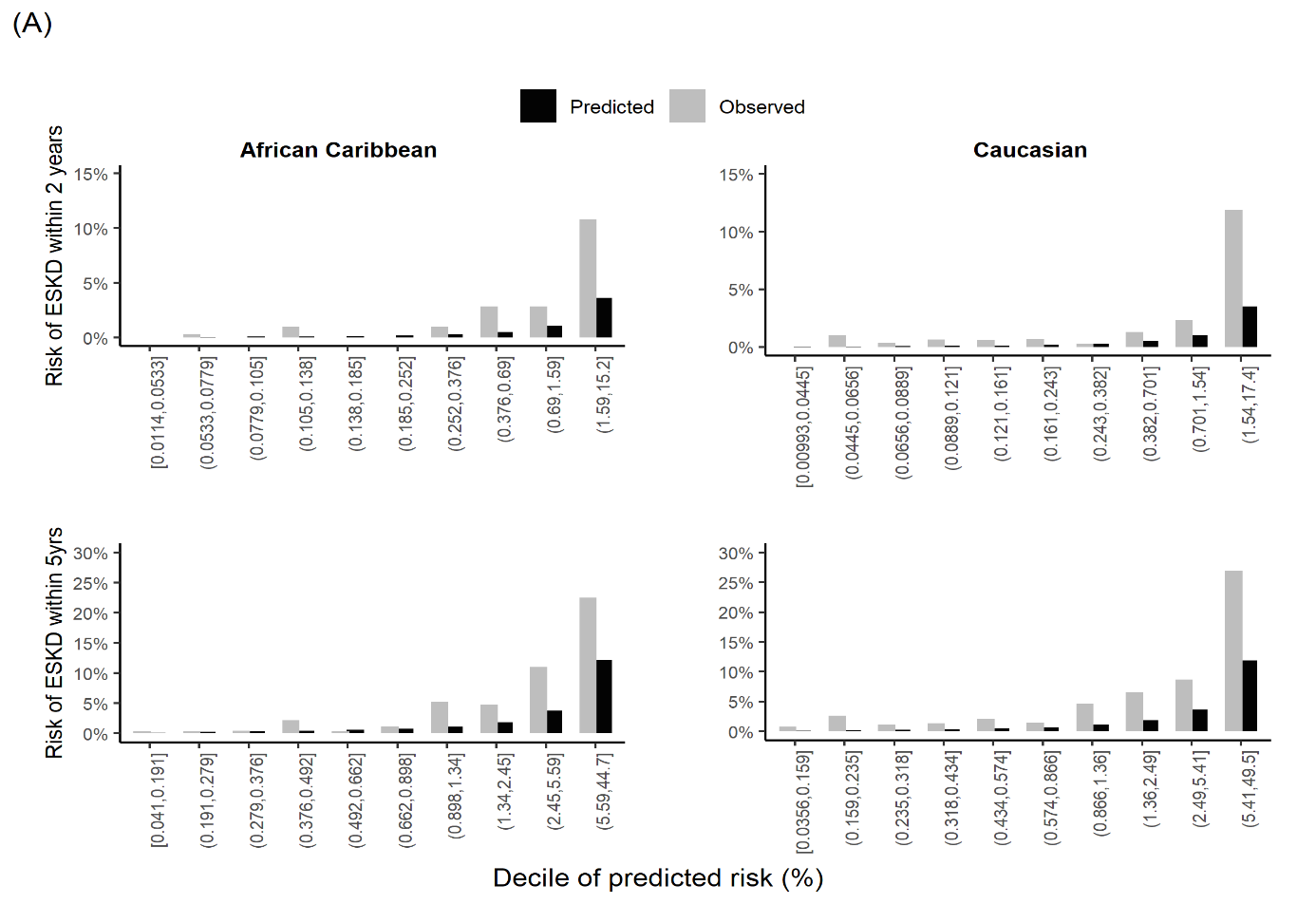


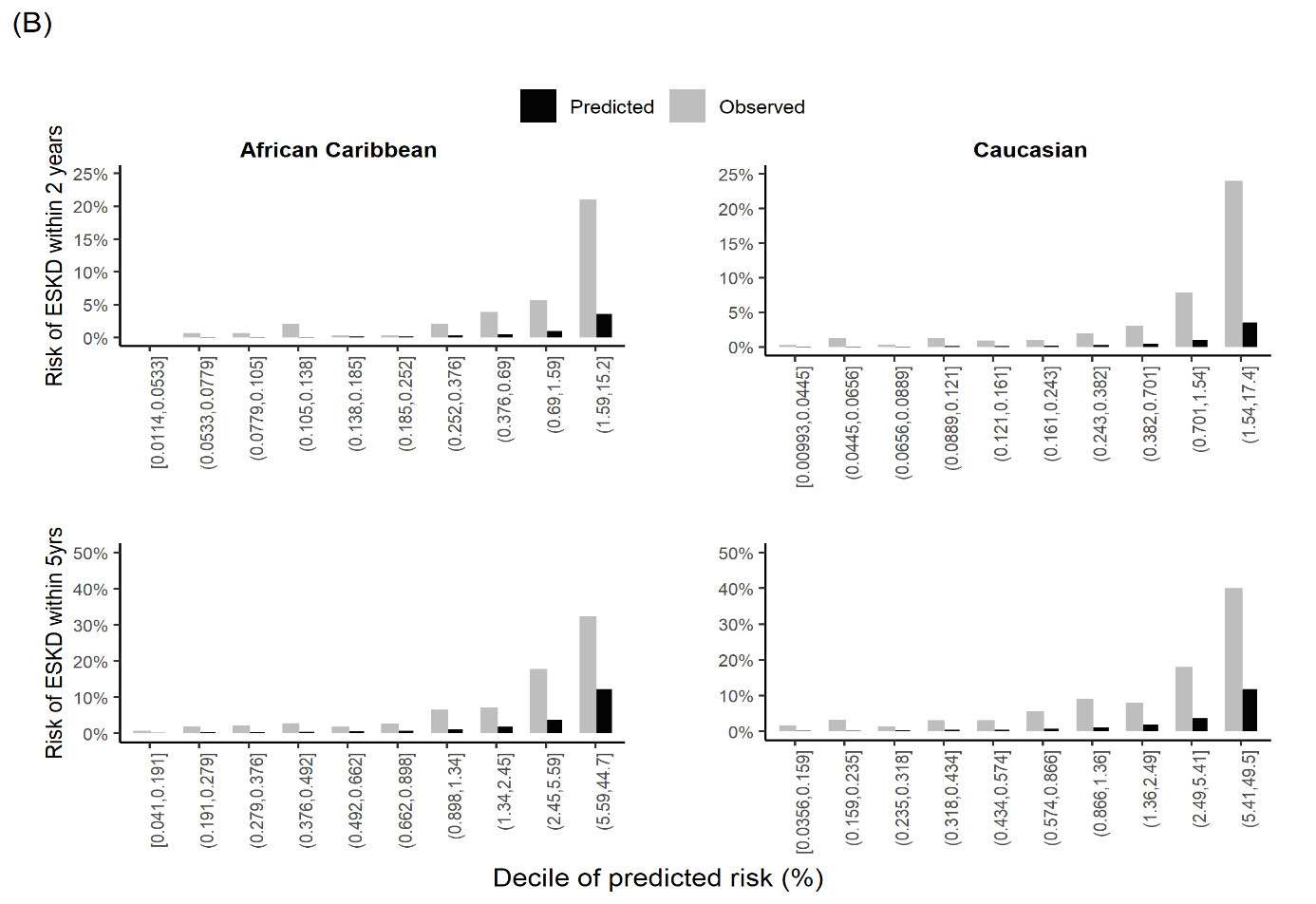


## **3. Sensitivity analyses in the complete case analysis (CCA) dataset. Results from the primary and secondary analyses**

Table S4: KFRE performance in African-Caribbean and Caucasian with T2DM and CKD.

|  | **2 years** | | | **5 years** | | |
| --- | --- | --- | --- | --- | --- | --- |
|  | **Discrimination** | | **Calibration** | **Discrimination** | | **Calibration** |
|  | **Harrell’C-index** | **Uno’C-index** | **ICI, E50, E90**  **(95% CI) )^¶^** | **Harrell’C-index** | **Uno’C-index** | **ICI, E50, E90**  **(95% CI)^¶^** |
| **Primary event defined as: EGFR<10ml/min** |  |  |  |  |  |  |
| **African-Caribbean in the CCA** | 0.845  (0.786, 0.904) | 0.845  (0.786, 0.904) | 0.012  (0.007, 0.018)  0.007  (0.004, 0.010)  0.028  (0.014, 0.045) | 0.837  (0.797, 0.876) | 0.837  (0.798, 0.876) | 0.024  (0.018, 0.031)  0.017  (0.012, 0.021)  0.057  (0.035, 0.078) |
| **Caucasian in the CCA** | 0.836  (0.765, 0.907) | 0.831  (0.757, 0.905) | 0.012  (0.007, 0.017)  0.006  (0.003, 0.008)  0.025  (0.014, 0.037) | 0.793  (0.743, 0.842) | 0.779  (0.726, 0.832) | 0.028  (0.021, 0.039)  0.016  (0.011, 0.024)  0.060  (0.043, 0.087) |
| **Secondary event defined: EGFR<15ml/min** |  |  |  |  |  |  |
| **African-Caribbean in the CCA** | 0.839  (0.794, 0.884) | 0.838  (0.793, 0.883) | 0.029  (0.020, 0.036)  0.020  (0.014, 0.024)  0.066  (0.044, 0.081) | 0.803  (0.767, 0.840) | 0.799  (0.792, 0.836) | 0.050  (0.041, 0.059)  0.038  (0.028, 0.046)  0.108  (0.081, 0.134) |
| **Caucasian in the CCA** | 0.853  (0.810, 0.896) | 0.851  (0.807, 0.894) | 0.036  (0.030, 0.043)  0.020  (0.015, 0.024)  0.080  (0.064, 0.098) | 0.801  (0.767, 0.835) | 0.786  (0.750, 0.822) | 0.069  (0.059, 0.082)  0.040  (0.034, 0.052)  0.152  (0.128, 0.190) |

CCA: Complete case analysis dataset. ICI: The integrated calibration index, the mean of Absolute Difference (AD) between observed and predicted risk. E50, E90 the median and the 90th percentile of ADs. ^¶^ non-parametric bootstrap 95% confidence limits of each of the ICI, E50 and E90 separately.

Figure S5: Kaplan Meier curves of ESKD-free probabilities by risk groups in the complete case analysis dataset. Risk groups defined as in the original KFRE development paper (predicted risk <3%, 3% to <5%, 5% to <15%, 15% to <25%, 25% to <50% and >=50%. A: predictions based on the primary event defined by sustained eGFR<10ml/min. B: predictions based on secondary event defined by sustained eGFR<15ml/min

| 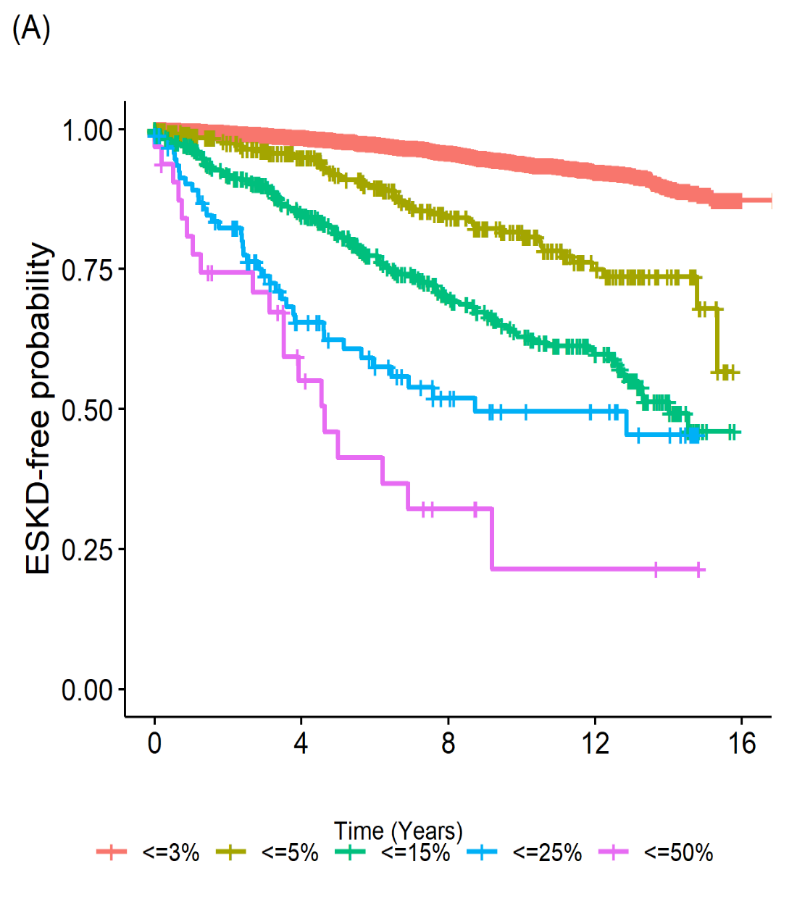 | 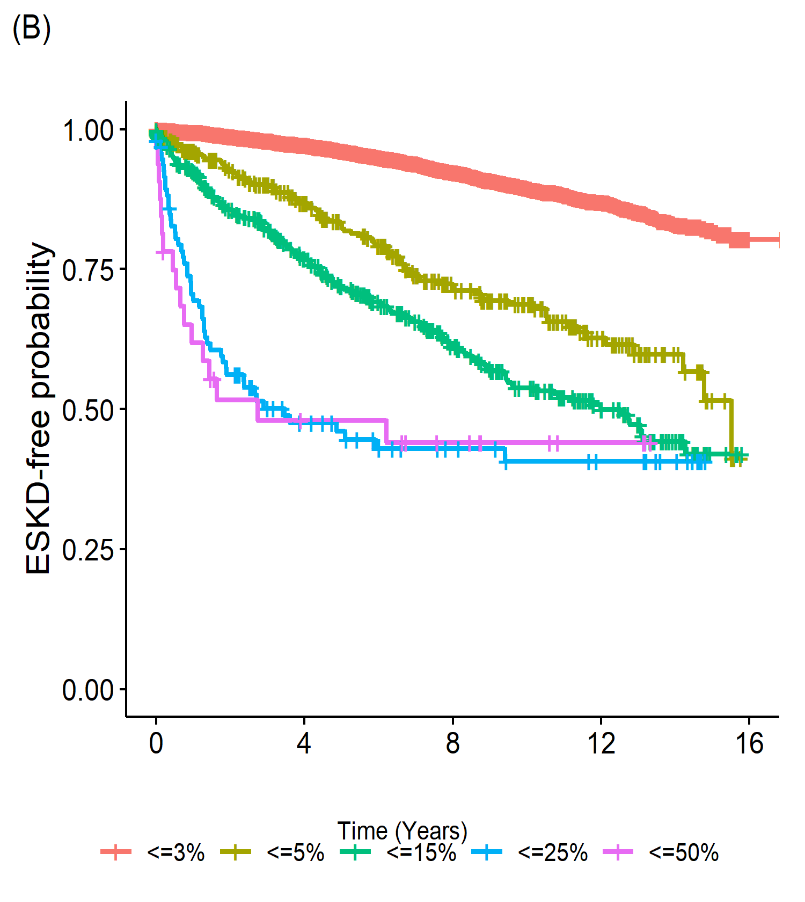 |
| --- | --- |

Figure S6: Observed and predicted ESKD risks for 2 and 5 years in the complete case analysis dataset. ESKD risks calculated in groups defined by decile of predicted risks. (A) with primary event defined as sustained eGFR<10 ml/min. (B) secondary event defined as sustained eGFR<15 ml/min


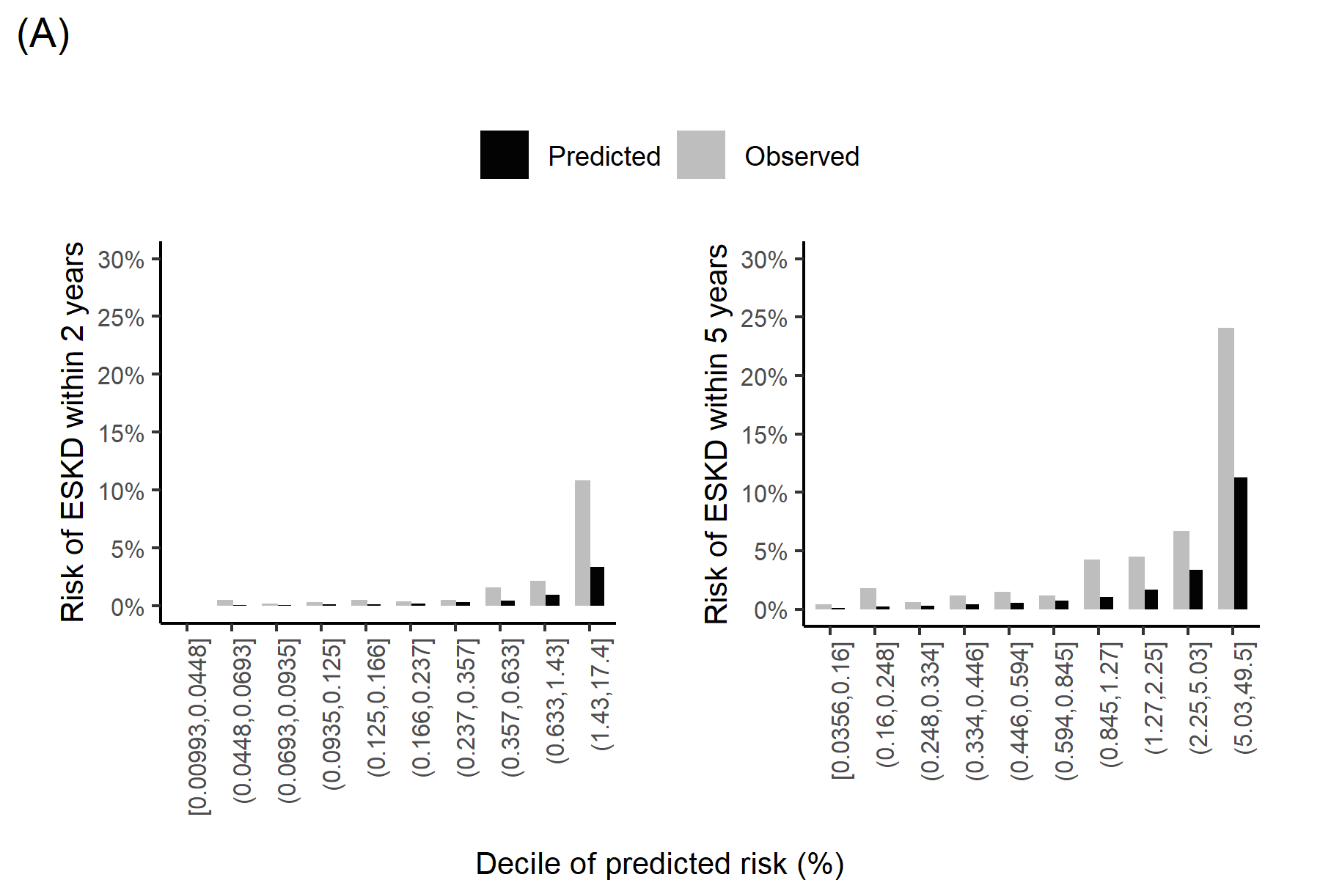


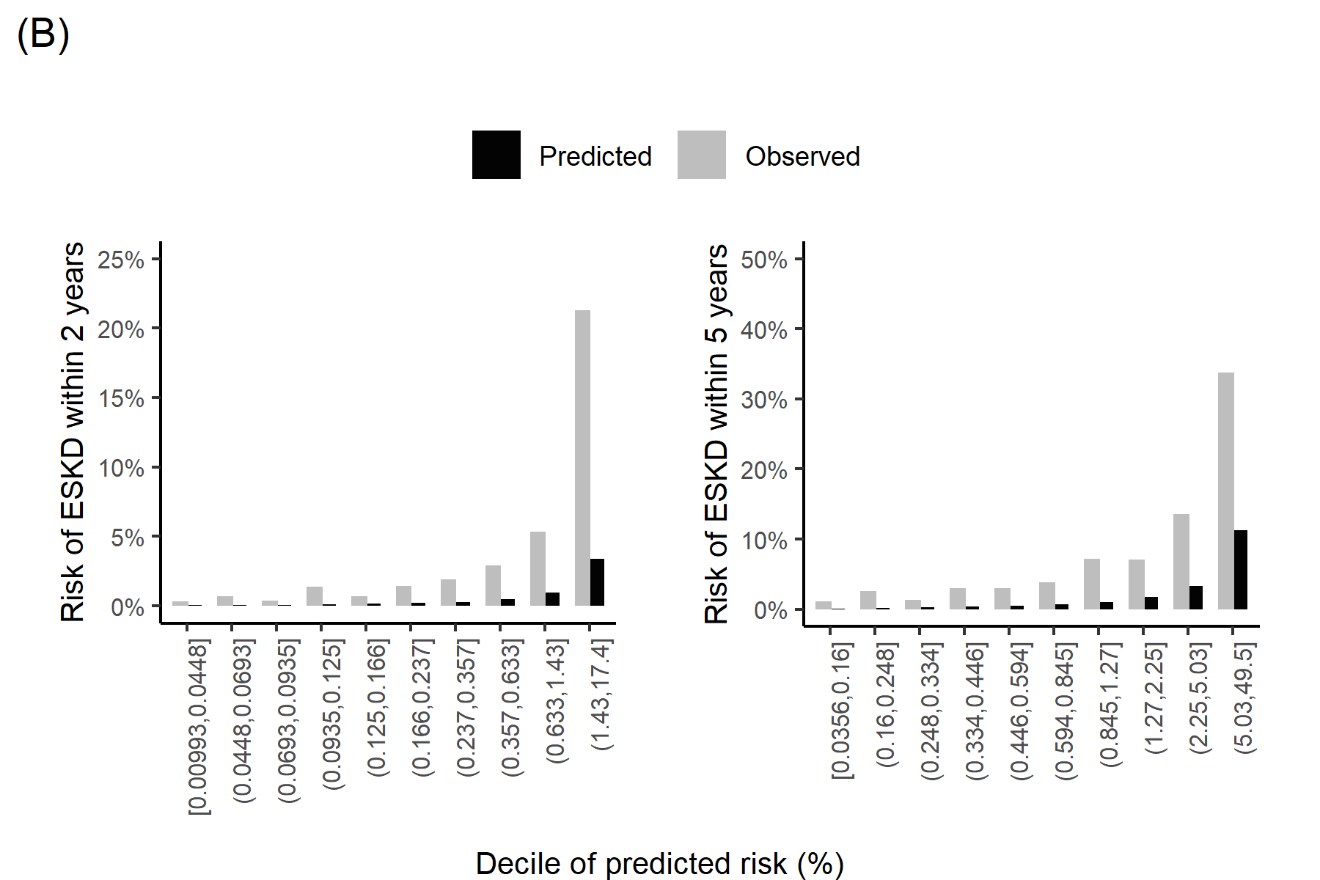


Figure S7: Kaplan Meier curves of ESKD-free probabilities by risk groups in each ethnicity group in the complete case analysis dataset. Risk groups defined as in the original KFRE development paper (predicted risk <3%, 3% to <5%, 5% to <15%, 15% to <25%, 25% to <50% and >=50%. A: predictions based on the primary event defined by sustained eGFR<10ml/min. B: predictions based on secondary event defined by sustained eGFR<15ml/min


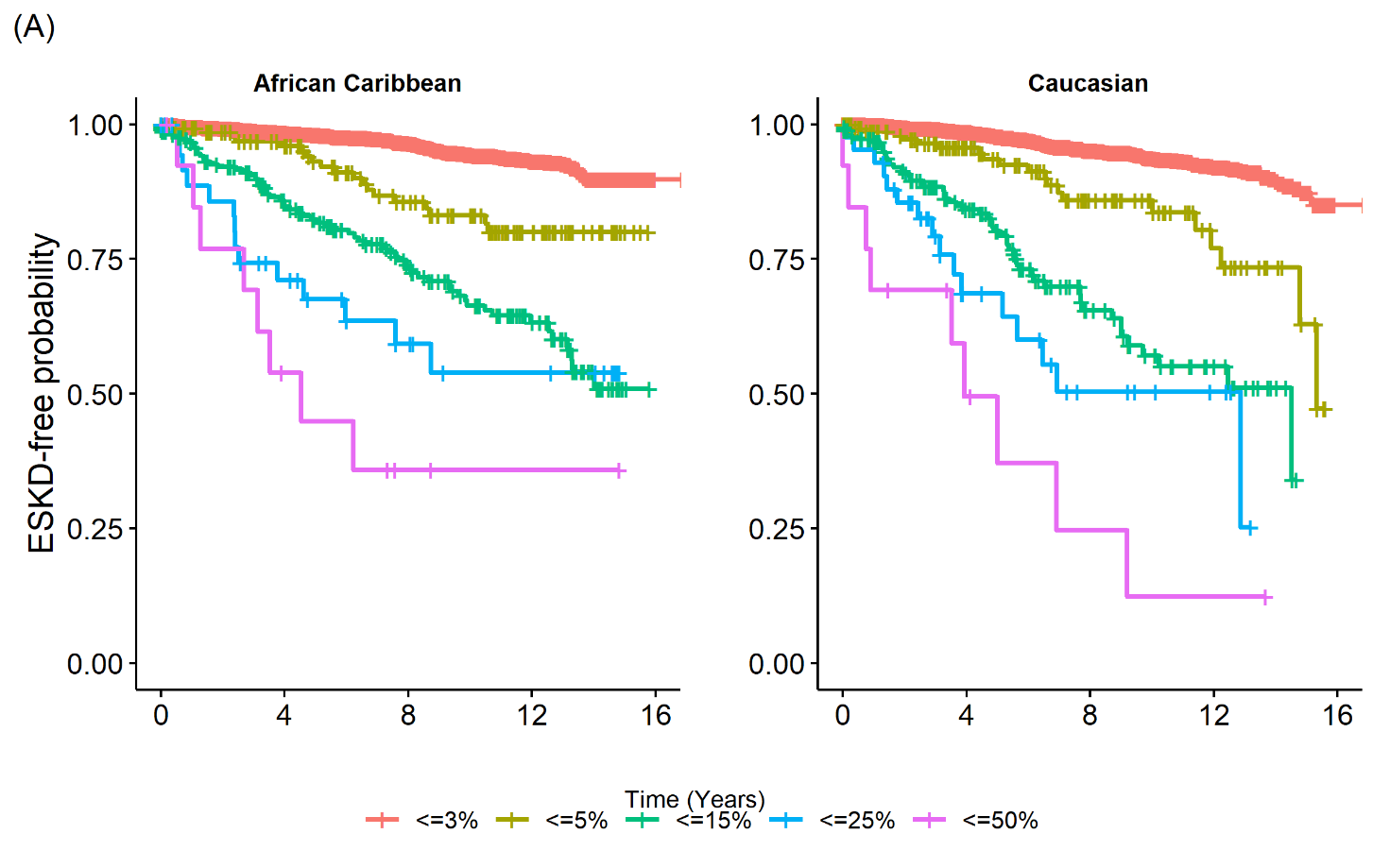


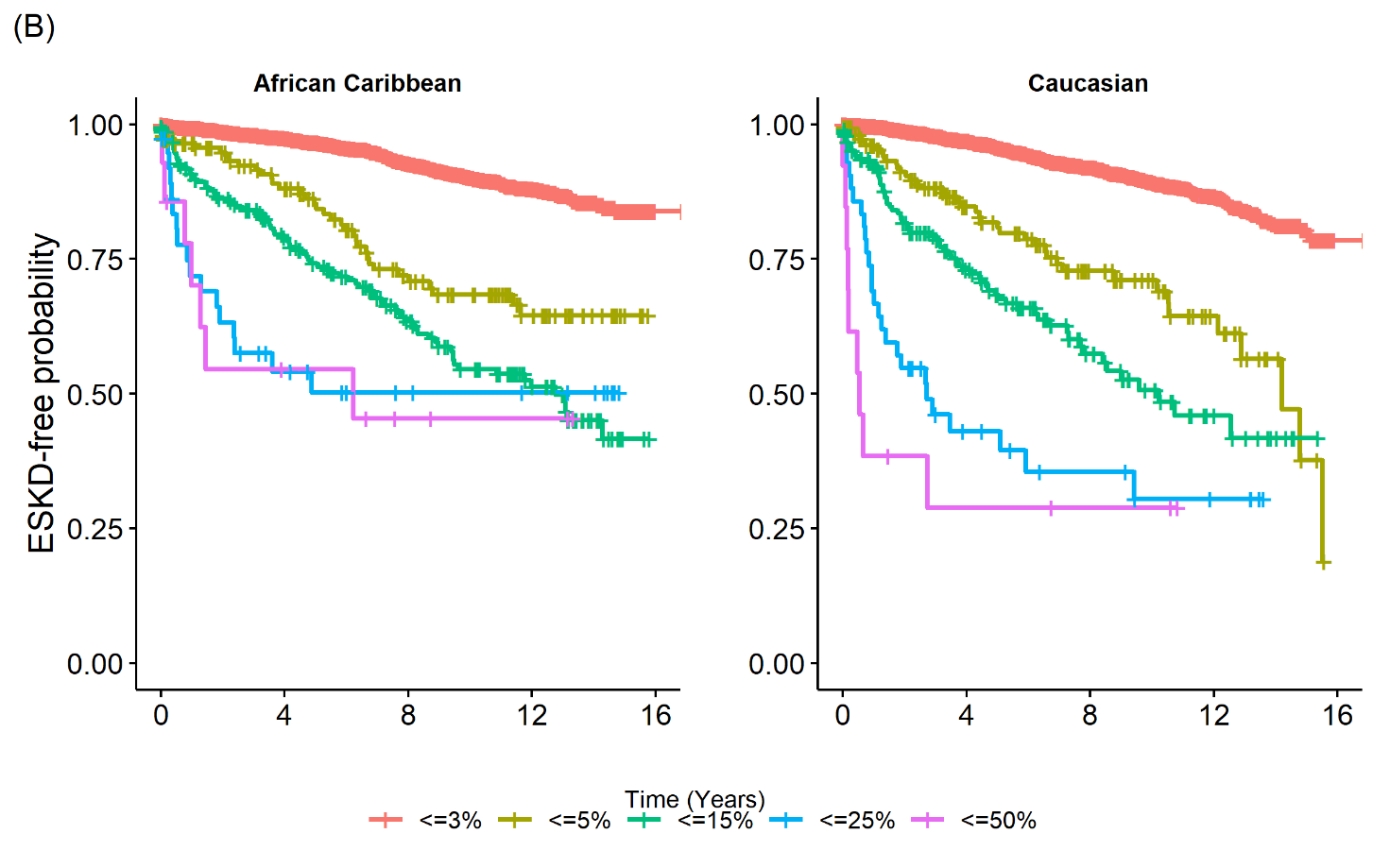


Figure S8: Observed and predicted ESKD risks for 2 and 5 years in each ethnicity group in the complete case analysis dataset. ESKD risks calculated in groups defined by decile of predicted risks. (A) with primary event defined as sustained eGFR<10 ml/min. (B) secondary event defined as sustained eGFR<15 ml/min.


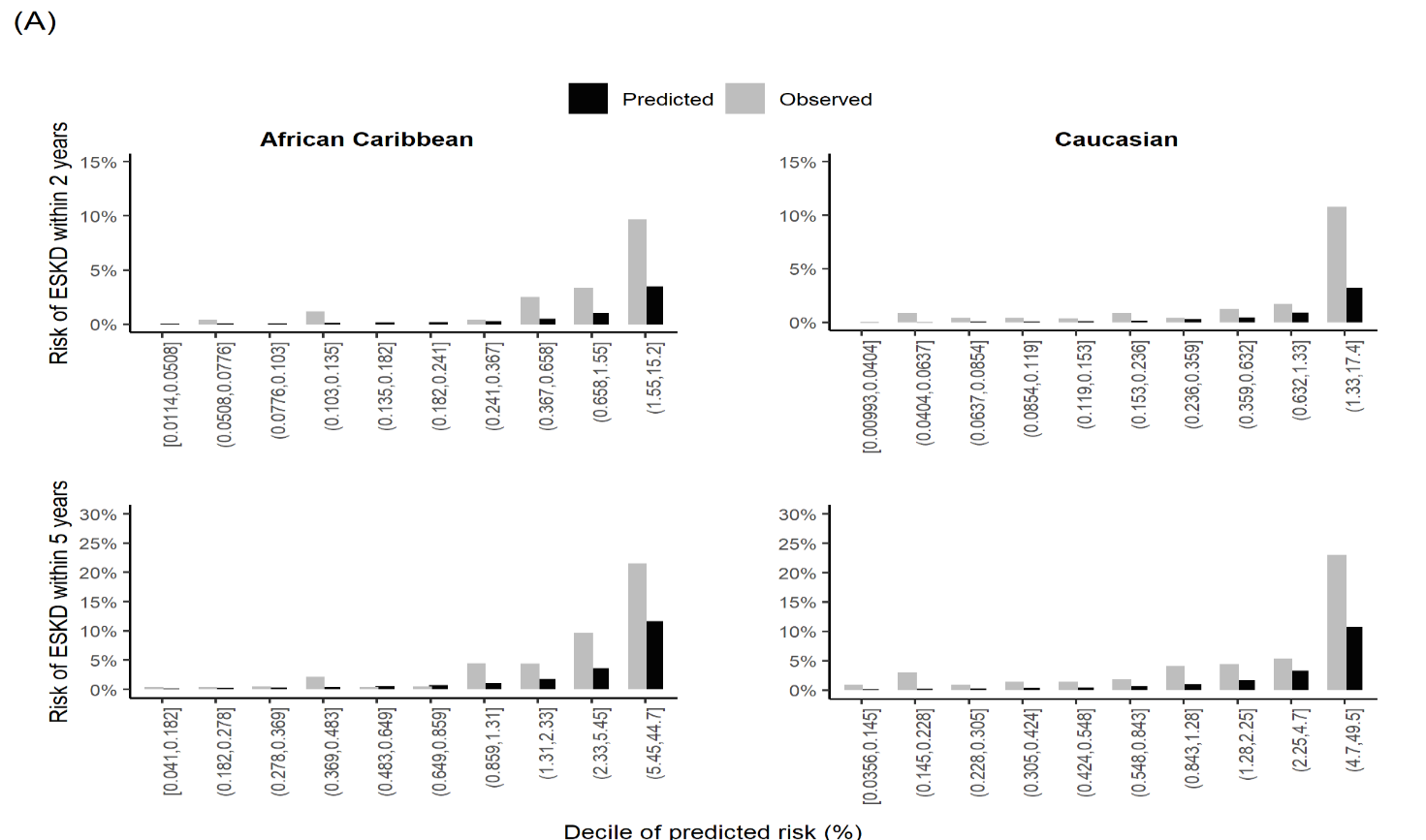


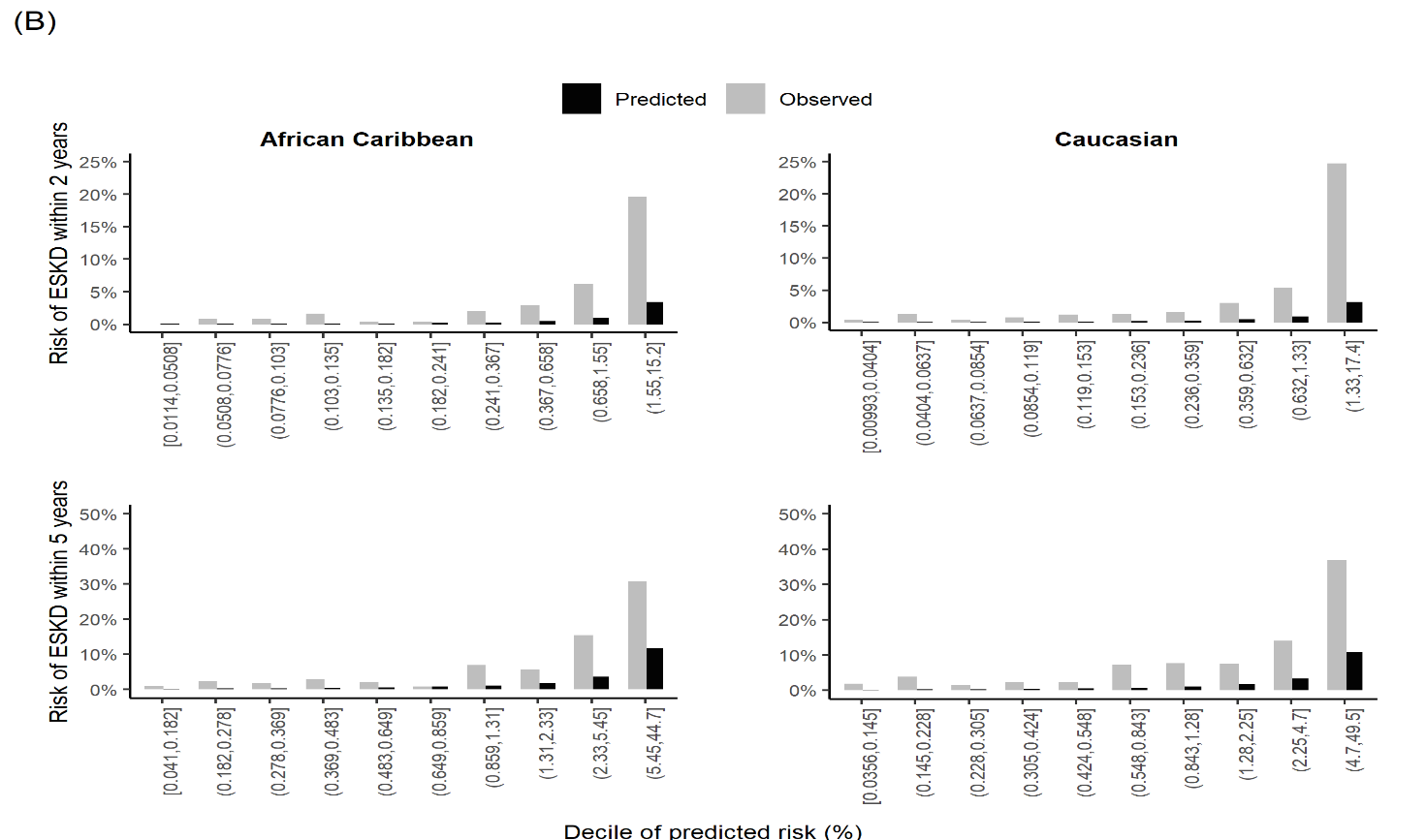


## **4. Recalibration of the original KFRE**

Recalibration was performed by adjusting the baseline risk in the KFRE model. The baseline risk describes the model’s predicted risk when all variables in the model are set as 0. In the KFRE, these values were centred around the mean values in the development cohort. The adjusted baseline risk for recalibration was calculated through post-estimation prediction of the survival function from the study cohort’s data using the primary endpoint (eGFR<10ml/min/1.73m^2^) as described elsewhere ^3^.

Table S5: Baseline survival in the original KFRE, the UK recalibrated KFRE and in the current cohort of people with T2DM and CKD

|  | **2-years** | **5-years** |
| --- | --- | --- |
| **Baseline survival** |  |  |
| Original model ^2^ | 0.9832 | 0.9365 |
| UK-recalibrated KFRE ^1^  (95% CI) | 0.9878  (0.9876, 0.9880) | 0.9570  (0.9563, 0.9576) |
| Recalibrated original KFRE with event as eGFR<10ml/min/1.73m^2^:  median[range]† | 0.9654  [0.9624, 0.9669] | 0.8998  [0.8912, 0.9044] |

†Median[range] of the baseline survival across the 40 imputation datasets.

Table S6: Calibration metrics with recalibrated KFRE in the full cohort using the primary ESKD event.

|  | **2-years** | **5-years** |
| --- | --- | --- |
|  | **ICI, E50, E90**  **(95% CI)^¶^** | **ICI, E50, E90**  **(95% CI)^¶^** |
| Full Cohort | 0.0062  (0.0045, 0.0079)  0.0051  (0.0033, 0.0069)  0.0057  (0.0023, 0.0091) | 0.0163  (0.0121, 0.0205)  0.0143  (0.0107, 0. 0179)  0.0155  (0.0091, 0.0219) |
| African-Caribbean | 0.0065  (0.0042, 0.0087)  0.0047  (0.0017, 0.0078)  0.0056  (0.0002, 0.0109) | 0.0164  (0.0112, 0.0216)  0.0111  (0.0060, 0.0163)  0.01600  (0.0015, 0.0304) |
| Caucasian | 0.0058  (0.0031, 0.0086)  0.0048  (0.0018, 0.0078)  0.0052  (0.000, 0.0105) | 0.0166  (0.0111, 0.0221)  0.0153  (0.0101, 0.0206)  0.0171  (0.0074, 0.0269) |

^¶^ non-parametric bootstrap 95% confidence limits of each of the ICI, E50 and E90 separately.

Figure S9: Kaplan Meier curves of ESKD-free probabilities by risk groups based on the recalibration of the original KFRE using the primary event.


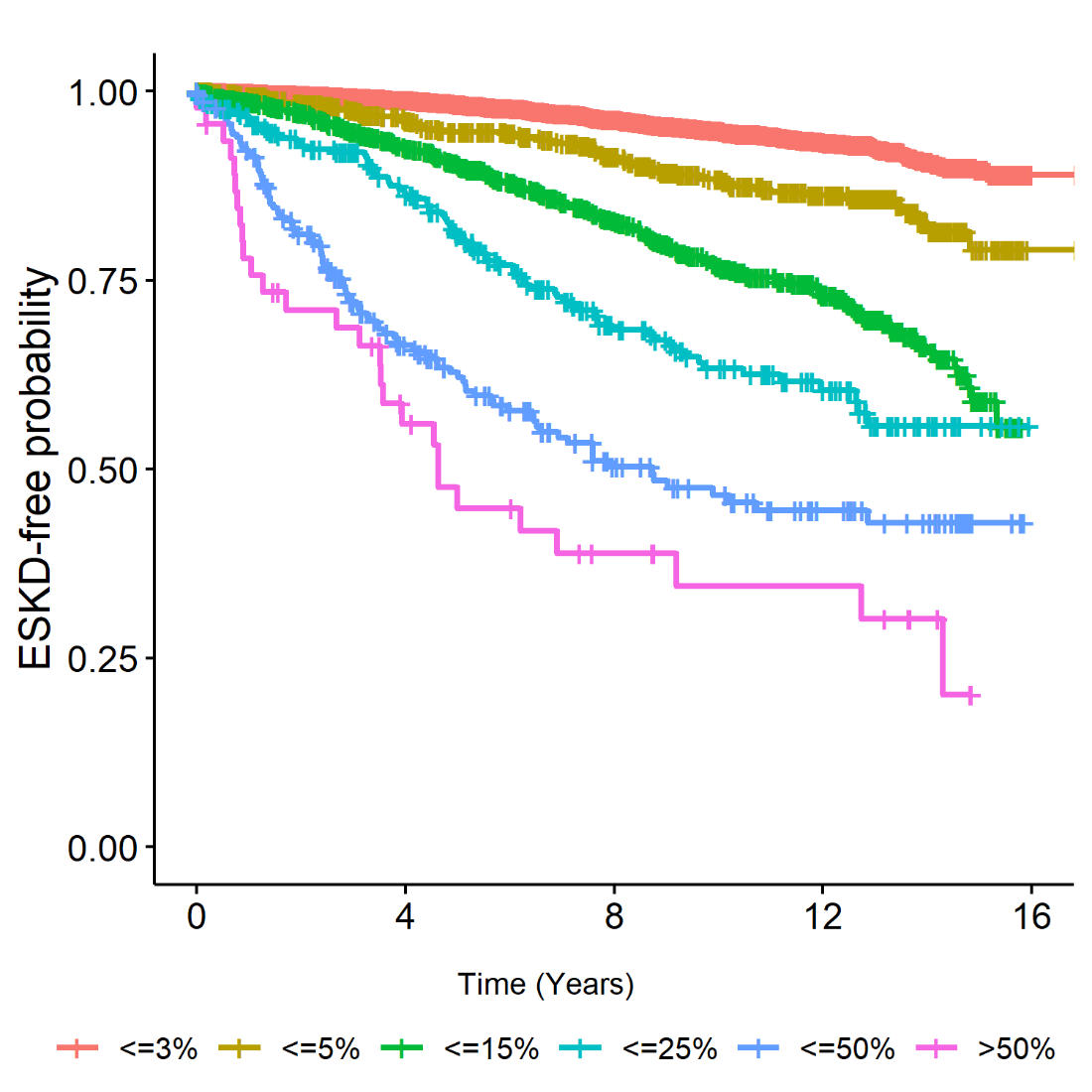


Figure S10: Observed and predicted primary ESKD risks for 2 and 5 years based on the recalibration of the original KFRE. ESKD risks calculated in groups defined by decile of predicted risks.


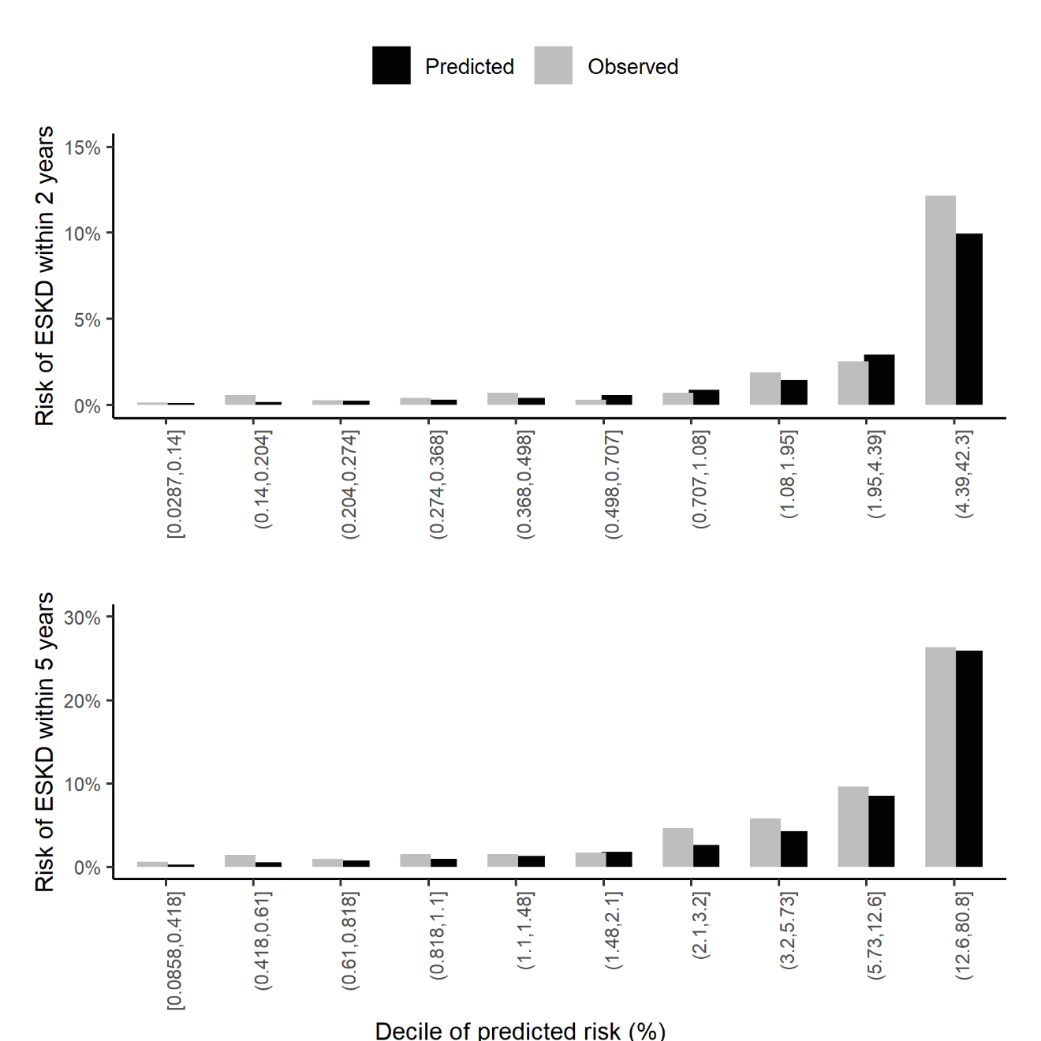


Figure S11: Kaplan Meier curves of ESKD-free probabilities by risk groups in each ethnicity group based on the recalibration of the original KFRE using the primary event.


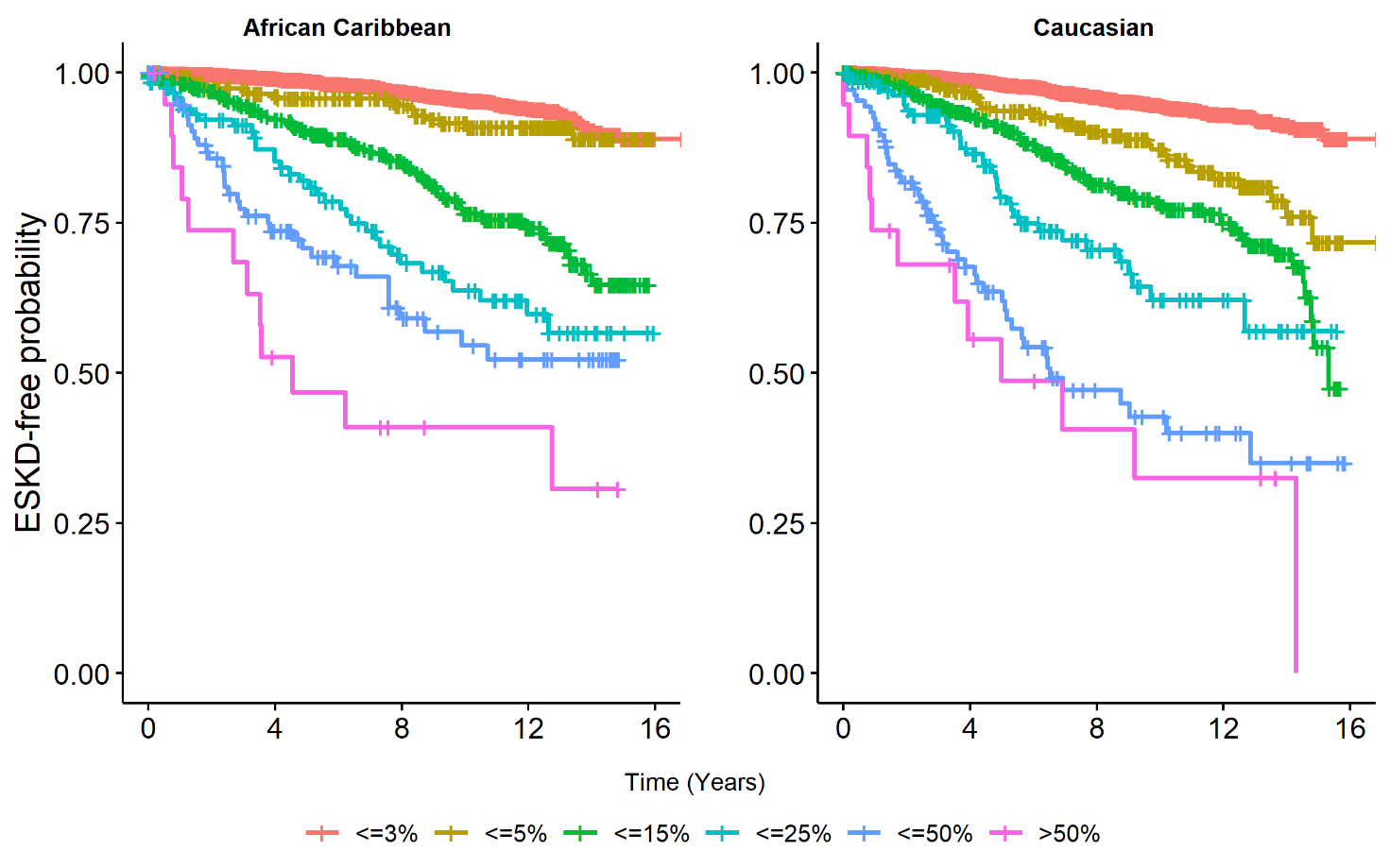


Figure S12: Observed and predicted ESKD risks for 2 and 5 years in each ethnicity group based on the recalibration of the original KFRE using the primary event. ESKD risks calculated in groups defined by decile of predicted risks.


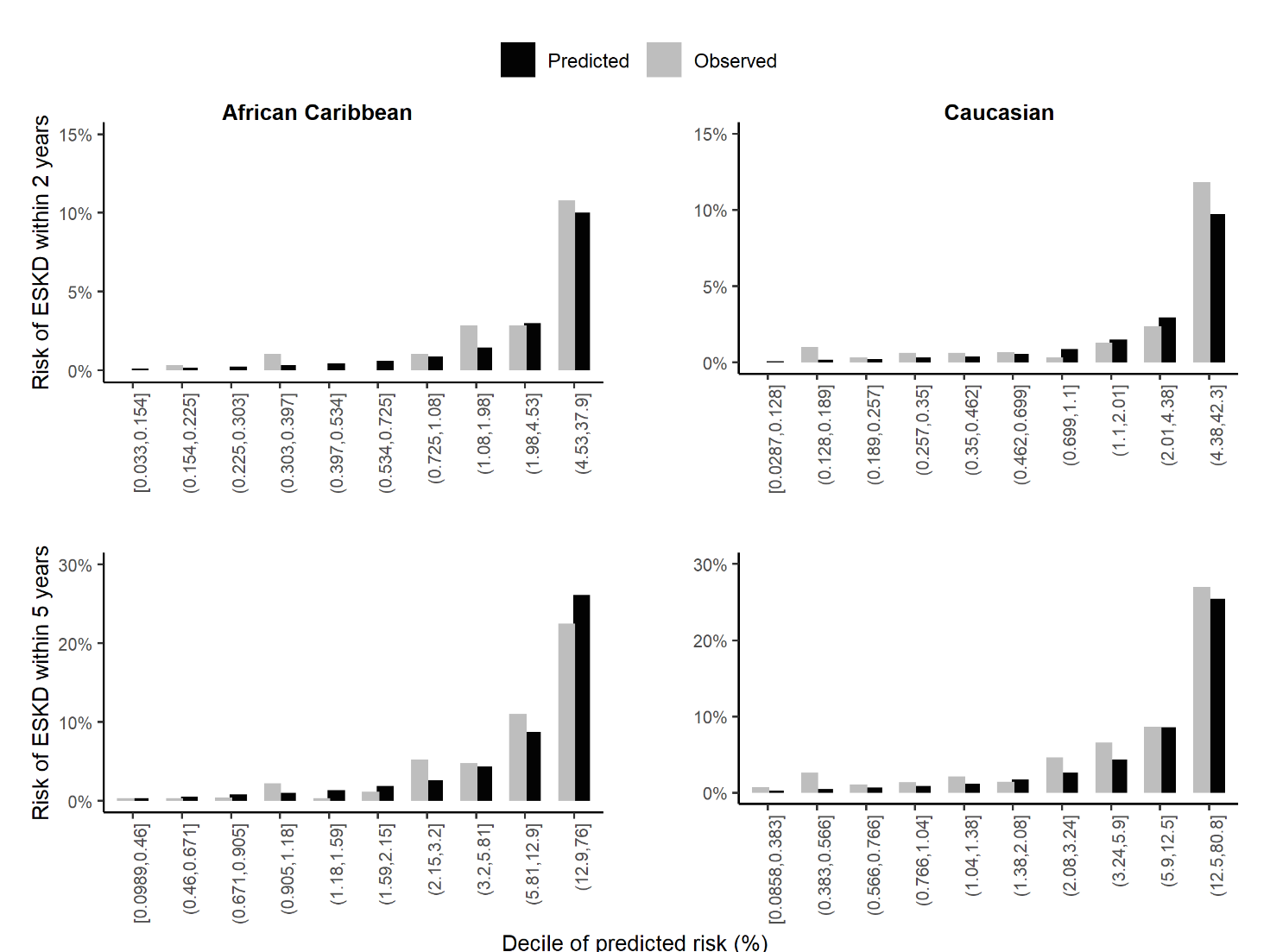


## **5. post-hoc analysis in the subgroup of individuals with an eGFR measurements<60ml/min within 2 years from the baseline**

Table S7: Baseline demographic and biochemical features of 7,296 people with T2DM and CKD and in the subgroup with a confirmatory eGFR<60ml/min within two years from the baseline.

|  | **Whole cohort**  **(N=7,296)** | **Subgroup with two eGFR within 2 years from the baseline* (N=6,249)** |
| --- | --- | --- |
| **Reached ESKD event (eGFR<10lm/min)** | 746 (10.2%) | 712 (11.4%) |
| **Reached ESKD event (eGFR<15lm/min)** | 1130 (15.5%) | 1067 (17.1%) |
| **Age** |  |  |
| Mean (SD) | 64.9 (11.9) | 65.8 (11.5) |
| Median (Q1, Q3) | 66.0 (57.0, 73.0) | 67.0 (58.0, 74.0) |
| **Ethnicity** |  |  |
| African Caribbean | 2955 (40.5%) | 2503 (40.1%) |
| Caucasian | 3298 (45.2%) | 2862 (45.8%) |
| Asian | 592 (8.1%) | 514 (8.2%) |
| Mixed | 199 (2.7%) | 170 (2.7%) |
| Other | 252 (3.5%) | 200 (3.2%) |
| **Gender** |  |  |
| Female | 2209 (30.3%) | 1867 (29.9%) |
| Male | 5087 (69.7%) | 4382 (70.1%) |
| **ACR (mg/mmol)** |  |  |
| N-Miss | 1366 | 1116 |
| Mean (SD) | 46.3 (45.7) | 47.9 (47.5) |
| Median (Q1, Q3) | 40.0 (18.7, 45.0) | 40.0 (19.5, 45.0) |
| **ACR (mg/mmol)†** |  |  |
| Mean (SD) | 46.8 (41.6) | 48.2 (43.5) |
| Median (Q1, Q3) | 41.0 (27.6, 47.0) | 41.0 (28.0, 47.2) |
| **eGFR (ml/min/1.73 m^2^)** |  |  |
| Mean (SD) | 45.2 (10.9) | 44.1 (11.0) |
| Median (Q1, Q3) | 47.6 (38.2, 54.2) | 46.1 (36.7, 53.1) |
| **eGFR stage** |  |  |
| G3a | 4226 (57.9%) | 3352 (53.6%) |
| G3b | 2203 (30.2%) | 2054 (32.9%) |
| G4 | 867 (11.9%) | 843 (13.5%) |

*Subgroup of people with a baseline eGFR between 15-59 ml/min/1.73m^2^. and a second confirmatory eGFR value between 15-59 ml/min/1.73m^2^ within 2 years from the baseline.

Table S8: KFRE performance in the subgroup with an eGFR<60 within 2 years from the baseline.

|  | **2 years** | | | **5 years** | | |
| --- | --- | --- | --- | --- | --- | --- |
|  | **Discrimination** | | **Calibration** | **Discrimination** | | **Calibration** |
|  | **Harrell’C-index** | **Uno’C-index** | **ICI, E50, E90**  **(95% CI) )^¶^** | **Harrell’C-index** | **Uno’C-index** | **ICI, E50, E90**  **(95% CI) )^¶^** |
| Primary event with eGFR<10 |  |  |  |  |  |  |
|  | 0.830  (0.824, 0.836) | 0.829  (0.823, 0.835) | 0.014  (0.010, 0.017)  0.009  (0.007, 0.010)  0.030  (0.022, 0.038) | 0.805  (0.801, 0.810) | 0.801  (0.797, 0.805) | 0.0390  (0.033, 0.045)  0.028  (0.022, 0.034)  0.081  (0.066, 0.095) |
| Secondary event with eGFR<15 |  |  |  |  |  |  |
|  | 0.831  (0.826, 0.835) | 0.829  (0.825, 0.834) | 0.014  (0.010, 0.017)  0.009  (0.007, 0.010)  0.030  (0.022, 0.038) | 0.793  (0.790, 0.796) | 0.786  (0.783, 0.789) | 0.075  (0.066, 0.084)  0.056  (0.039, 0.073)  0.153  (0.133, 0.174) |

*Data imputation was carried out and results were summarised. ICI: The integrated calibration index, the mean of Absolute Difference (AD) between observed and predicted risk. E50, E90 the median and the 90th percentile of ADs. ^¶^ non-parametric bootstrap 95% confidence limits of each of the ICI, E50 and E90 separately.

## **References**

1. Major RW, Shepherd D, Medcalf JF, Xu G, Gray LJ, Brunskill NJ. The Kidney Failure Risk Equation for prediction of end stage renal disease in UK primary care: An external validation and clinical impact projection cohort study. *PLoS Med*. 2019;16(11):e1002955.

2. Tangri N, Grams ME, Levey AS, et al. Multinational Assessment of Accuracy of Equations for Predicting Risk of Kidney Failure: A Meta-analysis. *JAMA*. 2016;315(2):164-74.

3. Sim J, Teece L, Dennis MS, Roffe C, Team SSS. Validation and Recalibration of Two Multivariable Prognostic Models for Survival and Independence in Acute Stroke. *PLoS One*. 2016;11(5):e0153527.
